# In Vitro Screening and Characterization of Feline-Derived Lactic Acid Bacteria as Potential Probiotic Candidates with Bile Salt Hydrolase Activity and Cholesterol-Removal Capacity

**DOI:** 10.3390/microorganisms14071466

**Published:** 2026-07-03

**Authors:** Yuqiang Zhang, Weiwei Wang, Huakai Wang, Qianqian Chen, Chengyi Miao, Shiqiang Zhu, Lishui Chen, Ran Wang, Wei Xiong

**Affiliations:** 1 Food Laboratory of Zhongyuan, Luohe 462300, China; zhangyuqiangyu@163.com (Y.Z.); bingzhi213608@163.com (W.W.); huakaiwhk@163.com (H.W.); 2020920269@stu.haut.edu.cn (Q.C.); miaochengyi@zyfoodlab.com (C.M.); sqzhu92@163.com (S.Z.); chlishui@sina.com (L.C.); 2 Key Laboratory of Precision Nutrition and Food Quality, Department of Nutrition and Health, China Agricultural University, Beijing 100083, China; wangran@cau.edu.cn

**Keywords:** feline obesity, feline-derived lactic acid bacteria, probiotic candidates, bile salt hydrolase, cholesterol removal, gastrointestinal tolerance, safety assessment

## Abstract

Feline obesity is an increasingly prevalent health concern and is closely associated with metabolic disorders and intestinal dysbiosis. This study aimed to screen feline-derived lactic acid bacteria (LAB) for bile salt hydrolase activity, in vitro cholesterol-removal capacity, and selected probiotic-associated characteristics. Approximately 700 LAB isolates were obtained from fecal samples of healthy domestic cats and evaluated for bile salt hydrolase (BSH) activity, among which 105 isolates were identified as BSH-positive. Further screening was performed based on BSH activity, cholesterol-removal capacity, adhesion-related properties, antioxidant activity, gastrointestinal tolerance, antibacterial activity, organic acid production, hemolytic activity, and antibiotic susceptibility. Three candidate strains were ultimately selected and preliminarily identified by 16S rRNA gene sequencing as *Enterococcus hirae* C283, *Ligilactobacillus animalis* C289, and *Enterococcus faecium* C422. These strains exhibited BSH activity and preliminary in vitro cholesterol-removal phenotypes under the tested culture conditions, together with tolerance to simulated gastrointestinal conditions, antioxidant activity, antibacterial activity, and organic acid production. No hemolytic activity was detected; however, all three strains exhibited resistant or intermediate phenotypes to multiple antibiotics, highlighting the need for further genome-based safety assessment, particularly for the two *Enterococcus* strains. Overall, these findings identified feline-derived LAB candidates with BSH activity, preliminary in vitro cholesterol-removal phenotypes, and multiple probiotic-associated characteristics. However, these in vitro findings do not demonstrate regulation of host lipid metabolism or blood lipid levels. Comprehensive safety assessment, particularly for the *Enterococcus* strains, and in vivo validation are required before further application.

## 1. Introduction

Obesity has become one of the most prevalent nutritional disorders in companion animals, with a particularly high and increasing incidence in domestic cats. Epidemiological studies estimate that approximately 45% of pet cats are overweight or obese [1], making obesity a major health concern in feline medicine [2]. Excessive adiposity not only impairs mobility and quality of life [3] but also predisposes cats to a range of metabolic disorders, including diabetes mellitus, dyslipidemia, and hepatic lipid accumulation [4]. Furthermore, obesity is frequently accompanied by oxidative stress and chronic low-grade inflammation, which may contribute to cellular damage and organ dysfunction [5]. Increasing evidence also indicates that obesity is associated with significant alterations in the composition and metabolic activity of the gut microbiota. Such obesity-related dysbiosis may disrupt bile acid metabolism, energy homeostasis, and host metabolic regulation, thereby further exacerbating metabolic abnormalities in cats [6].

Current strategies for managing feline obesity primarily rely on dietary energy restriction, increased physical activity, behavioral modification, and veterinarian-supervised weight-management programs [7]. Although these interventions can induce weight loss in some animals, their long-term effectiveness is often compromised by poor owner compliance, weight regain after treatment, and potential adverse effects on animal welfare and behavior [8]. Novel approaches, such as intragastric botulinum toxin administration, have also been explored; however, their safety, efficacy, and practical applicability remain insufficiently validated [9]. Consequently, there is a growing interest in developing alternative nutritional strategies that are safe, sustainable, and physiologically compatible with feline metabolism.

Among emerging nutritional interventions, probiotics have attracted considerable attention because of their ability to influence host metabolism through interactions with the intestinal microbiota. Studies in humans and experimental animal models have demonstrated that specific probiotic strains can improve lipid metabolism through multiple mechanisms, including modulation of gut microbial composition, enhancement of short-chain fatty acid production, activation of AMP-activated protein kinase (AMPK) signaling, and suppression of lipogenesis-related pathways involving sterol regulatory element-binding protein-1c (SREBP-1c) and fatty acid synthase (FAS) [10,11,12,13]. These effects have been associated with reduced serum cholesterol and triglyceride concentrations, improved insulin sensitivity, and attenuation of hepatic lipid accumulation, although evidence in cats remains limited.

Among the mechanisms underlying probiotic-mediated lipid regulation, bile salt hydrolase (BSH) activity has received particular attention. BSH catalyzes the deconjugation of bile salts, producing free bile acids with altered solubility and intestinal reabsorption, which may increase fecal bile acid excretion. This process promotes the conversion of cholesterol into newly synthesized bile acids in the liver, thereby contributing to cholesterol reduction [14]. In addition, deconjugated bile acids can act as signaling molecules that regulate metabolic pathways through receptors such as farnesoid X receptor (FXR) and Takeda G protein-coupled receptor 5 (TGR5), influencing both lipid and glucose metabolism [14]. Consequently, BSH activity has been widely regarded as an important functional marker for identifying probiotic strains with lipid-regulating potential. For example, *Lactobacillus paracasei* JY062 significantly reduced serum lipid levels and hepatic fat accumulation in high-fat diet-fed mice through modulation of the AdipoQ–AMPK–SREBP-1c signaling pathway and remodeling of the gut microbiota [15].

Despite these promising findings, the application of probiotics in companion animals has mainly focused on gastrointestinal health, immune regulation, and digestive disorders [16]. Studies specifically targeting feline obesity remain scarce. More importantly, most commercially available probiotics intended for cats are derived from human, dairy, or environmental sources rather than the feline gastrointestinal tract [17]. Host-derived bacterial strains may possess physiological characteristics associated with adaptation to the gastrointestinal environment of their original host species. However, isolation from a particular host does not itself demonstrate host-specific adhesion, persistence, colonization, or functional efficacy [18]. Given the distinctive gastrointestinal physiology and bile acid metabolism of cats [19], feline-derived LAB represent relevant microbial resources for further investigation, but their intestinal persistence and host-specific effects require direct validation in feline models.

Lactic acid bacteria (LAB) are among the most widely used probiotic microorganisms because of their favorable safety profiles and well-documented health-promoting properties [20]. Therefore, systematic screening of LAB derived from healthy feline hosts represents a rational strategy for identifying candidate strains for further probiotic evaluation. In the present study, BSH activity, in vitro cholesterol-removal capacity, gastrointestinal tolerance, adhesion-related characteristics, antioxidant activity, antibacterial activity, and preliminary safety characteristics were evaluated as in vitro screening parameters.

Therefore, the objective of the present study was to screen feline-derived LAB for BSH activity, in vitro cholesterol-removal capacity, and selected probiotic-associated characteristics. Candidate strains were further evaluated for gastrointestinal tolerance, adhesion-related surface properties, antioxidant activity, antibacterial activity, organic acid production, hemolytic activity, and antibiotic susceptibility. The novelty of this study lies in the application of a stepwise in vitro screening strategy centered on BSH activity and cholesterol-removal capacity to a collection of feline-derived LAB. The selected isolates provide candidates for further mechanistic, safety, and in vivo evaluation.

## 2. Materials and Methods

### 2.1. Origin of the Feline-Derived LAB Strain Collection

An established collection of approximately 700 feline-derived LAB isolates was used in the present study. These isolates had been previously recovered from fecal samples collected from 300 clinically healthy individual domestic cats in Luohe, Henan Province, China, between June and December 2023. The fecal samples were obtained from different pet stores, veterinary hospitals, cat cafés, and private breeding households. Only one fecal sample was collected from each cat, and repeated sampling from the same individual was avoided. The sampled cats included more than ten common domestic cat breeds, such as British Shorthair, Ragdoll, Exotic Shorthair, Chinese domestic cats, and other domestic breeds. Healthy cats were defined as animals showing no obvious clinical signs of disease, no diarrhea or abnormal fecal appearance at the time of sampling, no history of chronic gastrointestinal disorders, and no antibiotic treatment within the previous three months. Because the samples were collected from multiple non-laboratory sources, complete metadata on age, sex, and spay/neuter status were not available for all cats. The original sample collection, isolation procedure, preliminary phenotypic characterization, and preservation of this strain collection have been described previously [21]. No new feline fecal samples were collected for the present study. In the present work, the preserved isolates were revived from frozen stocks and subjected to a new stepwise in vitro screening workflow centered on BSH activity and cholesterol-removal capacity.

### 2.2. Revival and Preparation of LAB Isolates for Functional Screening

Before functional screening, the preserved LAB isolates were revived from −80 °C glycerol stocks by inoculation into de Man–Rogosa–Sharpe (MRS) broth and incubation at 37 °C for 24 h under anaerobic conditions. Each isolate was subcultured twice under the same conditions to ensure adequate viability and metabolic activity before subsequent assays. The functional experiments described below were newly performed for the present study. No previously published functional data were reused.

### 2.3. Preliminary Screening and Semi-Quantitative Evaluation of Bile Salt Hydrolysis

The preliminary screening of bile salt hydrolase (BSH)-producing strains was performed according to Dong et al. [22], whereas semi-quantitative evaluation of bile salt hydrolysis was conducted according to Guo et al. [23], with minor modifications. For qualitative screening, LAB isolates were inoculated onto modified MRS agar supplemented with 0.5% sodium glycodeoxycholate (GDCA) and 0.037% CaCl_2_ and incubated anaerobically at 37 °C for 48 h. Colonies surrounded by visible precipitation zones were considered BSH-positive strains.

For semi-quantitative evaluation, selected BSH-positive strains were cultured in MRS broth at 37 °C for 24 h. Bacterial cells were collected by centrifugation, washed twice with phosphate-buffered saline (PBS), and resuspended to the same bacterial density based on OD600 to reduce the influence of biomass differences among strains. The bacterial suspensions were disrupted by ultrasonication on ice at 300 W for 10 min using intermittent pulses of 5 s on and 5 s off. After centrifugation at 12,000 rpm at 4 °C for 10 min, the supernatants were collected as crude enzyme extracts. The crude enzyme extracts were incubated with sodium glycodeoxycholate as the substrate at 37 °C for 30 min. After incubation, the released glycine was detected using the ninhydrin colorimetric method. Absorbance was measured at 570 nm, and bile salt hydrolysis percentage (%) was calculated as a semi-quantitative screening index. Protein concentration of the crude enzyme extracts was not determined in the present screening assay; therefore, the results were not expressed as standardized BSH activity units such as U/mg protein. Accordingly, the reported values should be interpreted as bile salt hydrolysis percentages rather than protein-normalized enzyme activities.

### 2.4. Determination of Cholesterol-Removal Capacity

The cholesterol-removal ability of LAB strains was determined according to Chabi et al. [24], with minor modifications. Briefly, LAB strains were inoculated into MRS broth supplemented with cholesterol (100 μg/mL) and bile salts (0.3%) and incubated anaerobically at 37 °C for 48 h. After centrifugation, the residual cholesterol concentration in the supernatant was determined using the sulfuric acid–glacial acetic acid–ferrous sulfate colorimetric method. Cholesterol-removal efficiency was calculated as follows:

Cholesterol removal (%) = [(C_0_ − C_1_)/C_0_] × 100   (1)

where C_0_ represents the cholesterol concentration of the uninoculated control and C_1_ represents the residual cholesterol concentration after incubation.

### 2.5. Physiological and Biochemical Characteristics

The physiological and biochemical characteristics of selected LAB strains were evaluated according to Zhang et al. [25]. Growth was examined under different pH conditions (3.0–10.0), NaCl concentrations (3.0% and 6.5%), and temperatures (5, 10, 45, and 50 °C). Bacterial growth was assessed by measuring optical density at 600 nm (OD_600_).

Cell-surface hydrophobicity and auto-aggregation ability were determined according to Wang et al. [26]. Hydrophobicity and auto-aggregation percentages were calculated based on changes in OD_600_ values.

### 2.6. Determination of Growth and Acid-Production Curves

Selected LAB strains were inoculated into MRS broth and incubated at 37 °C. Optical density at 600 nm (OD_600_) and viable cell counts were measured every 2 h during a 24 h cultivation period. The pH values of fermentation broths were recorded every 6 h for up to 48 h to evaluate acid-production capacity.

### 2.7. In Vitro Antioxidant Activity

The antioxidant activity of LAB strains was evaluated according to Düz et al. [27], Sanna and Fadda [28], and Wu et al. [29]. Overnight cultures were harvested by centrifugation, washed twice with PBS, and resuspended to an OD_600_ of approximately 1.0.

DPPH radical-scavenging activity and superoxide anion radical-scavenging activity were determined according to Düz et al. [27]. Hydroxyl radical-scavenging activity was evaluated according to Sanna and Fadda [28]. ABTS radical-scavenging activity was determined according to Wu et al. [29]. Radical-scavenging activities were calculated based on absorbance changes relative to the corresponding control groups.

### 2.8. In Vitro Tolerance to Simulated Gastric and Intestinal Fluids

Tolerance to simulated gastric fluid (SGF) and simulated intestinal fluid (SIF) was evaluated according to Zhang et al. [30]. SGF (pH 2.5) consisted of 0.35 g pepsin dissolved in 100 mL of sterile 0.2% NaCl solution. SIF (pH 8.0) consisted of 0.1 g trypsin, 1.1 g sodium bicarbonate, and 1.8 g bile salts dissolved in 100 mL of sterile 0.2% NaCl solution.

LAB strains were exposed to SGF for 3 h and SIF for 4 h. Viable counts were determined before and after treatment, and survival rates were calculated accordingly.

### 2.9. 16S rRNA Gene Analysis

Genomic DNA was extracted from bacterial cultures and used as templates for PCR amplification. Universal bacterial primers 27F (5′-AGAGTTTGATCCTGGCTCAG-3′) and 1492R (5′-GGTTACCTTGTTACGACTT-3′) were used to amplify the 16S rRNA gene. PCR products were sequenced by Sangon Biotech Co., Ltd. (Shanghai, China). Sequence similarity analysis was performed using the online NCBI BLASTn program against the GenBank database (https://blast.ncbi.nlm.nih.gov/Blast.cgi; accessed on 20 April 2025). Phylogenetic trees were constructed using MEGA-X software (version 10.1.5) based on the neighbor-joining method.

### 2.10. Preliminary Safety Assessment of LAB Strains

Hemolytic activity was evaluated using Columbia blood agar plates. Fresh cultures of strains C283, C289, and C422 were streaked onto blood agar plates and incubated at 37 °C for 24–48 h. *Staphylococcus aureus* ATCC 29213^T^ was used as the positive control. Hemolytic activity was assessed by observing the presence or absence of hemolytic zones surrounding bacterial colonies.

Antibiotic susceptibility was determined using a modified disk diffusion method according to Niu et al. [31]. The antibiotics tested included gentamicin (GEN, 10 μg/disk), ciprofloxacin (CIP, 5 μg/disk), ceftriaxone (CTR, 30 μg/disk), erythromycin (E, 15 μg/disk), ampicillin (AMP, 10 μg/disk), tetracycline (TET, 30 μg/disk), compound sulfamethoxazole (SXT, 25 μg/disk), chloramphenicol (C, 30 μg/disk), lincomycin (MY, 2 μg/disk), and penicillin (PEN, 10 μg/disk). Inhibition-zone diameters were interpreted according to the criteria proposed by de Souza et al. [32]. Because the selected isolates belonged to different genera, the disk diffusion assay was used only as a preliminary phenotypic screening method. The results were interpreted cautiously and were not regarded as definitive antimicrobial resistance classifications. Further MIC-based testing using appropriate genus- or species-specific cut-off values is required for comprehensive safety assessment.

### 2.11. Determination of Antibacterial Activity

Broad-spectrum antibacterial activity was evaluated according to the agar well diffusion method described by Sirichokchatchawan et al. [33] with minor modifications. Indicator strains included *Pseudomonas aeruginosa* CICC 23694^T^, *Staphylococcus aureus* ATCC 29213^T^, *Listeria monocytogenes* CICC 23929^T^, *Escherichia coli* CICC 24189^T^, *Bacillus subtilis* CICC 10275^T^, and *Shigella dysenteriae* CICC 23829^T^.

After incubation at 37 °C for 24 h, inhibition-zone diameters were measured and recorded.

### 2.12. Preliminary Identification of Antibacterial Substances

To further clarify the potential contributors to the antibacterial activity observed in the agar well diffusion assay, the effects of organic acids, hydrogen peroxide, and proteinaceous antimicrobial compounds were evaluated using cell-free fermentation supernatants of strains C283, C289, and C422. Overnight cultures were centrifuged at 8000 rpm for 10 min, and the supernatants were collected for subsequent treatments.

To evaluate the contribution of organic acids and low pH, the pH values of the fermentation supernatants were adjusted to 6.5–7.0 using sterile 1 M NaOH. Untreated acidic supernatants were used as controls. To assess the role of hydrogen peroxide, the supernatants were treated with catalase at a final concentration of 0.5 mg/mL and incubated at 37 °C for 2 h. To determine whether proteinaceous antimicrobial substances were involved, neutralized supernatants were treated separately with proteinase K, trypsin, and pepsin at a final concentration of 1 mg/mL and incubated at 37 °C for 2 h. After each treatment, the samples were centrifuged, and the resulting supernatants were tested against *Escherichia coli* CICC 24189^T^ using the agar well diffusion method described above. Untreated MRS broth was used as the negative control. Each assay was performed in triplicate.

### 2.13. Determination of Organic Acid Contents by UPLC–Orbitrap–MS

Organic acid contents in fermentation broths were determined using ultra-performance liquid chromatography coupled with Orbitrap mass spectrometry (UPLC–Orbitrap–MS) after 3-nitrophenylhydrazine (3-NPH) derivatization. Briefly, fermentation broths of C283, C289, and C422 were centrifuged at 12,000 rpm at 4 °C for 10 min, and the supernatants were collected. Uninoculated MRS broth incubated under the same conditions was processed in parallel as the medium blank, and procedural blanks prepared with ultrapure water were also included.

For sample pretreatment, 1 mL of methanol–chloroform solution (7:3, *v*/*v*) was added to each sample and extracted on ice for 30 min. Then, 600 μL of ultrapure water was added, followed by centrifugation at 12,000 rpm at 4 °C for 10 min. The upper aqueous phase was collected and derivatized with 10 μL of 0.1 M 1-ethyl-3-(3-dimethylaminopropyl) carbodiimide and 10 μL of 0.1 M 3-NPH at 40 °C for 30 min. Chromatographic separation was performed using a BEH C18 column (50 × 2.1 mm, 1.8 μm) supplied by Waters (Milford, MA, USA) at 40 °C. The flow rate was 0.35 mL/min, and the injection volume was 2 μL. The mobile phases were water containing 0.1% formic acid and acetonitrile containing 0.1% formic acid. High-resolution mass spectrometric analysis was conducted using a Q Exactive hybrid Q-Orbitrap mass spectrometer (Thermo Fisher Scientific, Waltham, MA, USA) in negative electrospray ionization mode. Organic acids were quantified using external standard curves prepared from authentic standards of lactic acid, acetic acid, citric acid, and succinic acid. Quality-control samples were prepared by pooling representative sample extracts and injected periodically during the analytical sequence. The organic acid concentrations detected in the uninoculated MRS blank were subtracted from the corresponding fermented samples before statistical analysis.

### 2.14. Statistical Analysis

All experiments were performed using three independent biological replicates for each strain or treatment group. For bacterial assays, each biological replicate was prepared from an independently cultured bacterial suspension on a separate experimental occasion. Each assay was conducted in technical triplicate to reduce analytical variation. Data are presented as the mean ± standard deviation (SD). Statistical analyses were performed using SPSS software version 25.0 (IBM Corp., Armonk, NY, USA). Data normality and homogeneity of variance were assessed using the Shapiro–Wilk test and Levene’s test, respectively, before parametric analysis. Percentage data, including cholesterol-removal efficiency, survival rate, cell-surface hydrophobicity, auto-aggregation, and radical-scavenging activity, were arcsine-square-root transformed when necessary before statistical analysis. For comparisons among different strains or treatment groups, one-way analysis of variance (ANOVA) followed by Tukey’s multiple-comparison test was used. For time-course data, including growth curves, viable cell counts, and acid-production curves, two-way ANOVA was used to evaluate the effects of strain, time, and their interaction. When significant interactions were detected, differences among strains at the same time point were further analyzed using one-way ANOVA followed by Tukey’s test. Differences were considered statistically significant at *p* < 0.05.

## 3. Results

### 3.1. Established Strain Collection Used for the Present Screening Workflow

The present study used an established collection of approximately 700 feline-derived LAB isolates that had been previously isolated, preliminarily characterized, and preserved by our group [21]. The original sampling, isolation procedure, and preliminary taxonomic characterization are not presented as new findings in this study. The first newly conducted step in the present screening workflow was the qualitative evaluation of BSH activity. Subsequent quantitative BSH analysis, cholesterol-removal assays, and multi-parameter functional characterization were also newly performed using revived isolates from the preserved strain collection.

### 3.2. Preliminary Screening of BSH-Positive Strains

BSH activity was used as the primary criterion for the initial functional screening of 700 LAB isolates. A total of 105 isolates were identified as BSH-positive based on the formation of precipitation halos on MRS agar supplemented with GDCA.

**Figure 1:**
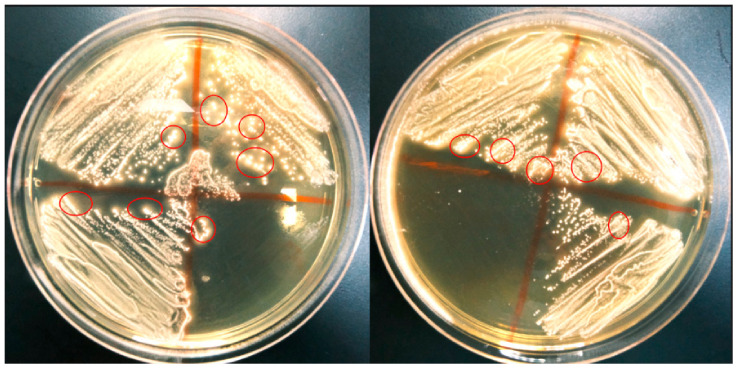
Preliminary screening of BSH-positive LAB isolates. Red circles indicate visible precipitation halos around colonies on GDCA-containing MRS agar, which were used as the criterion for identifying BSH-positive isolates.

As shown in Figure 1, BSH-positive isolates formed milky-white precipitation halos around the colonies after anaerobic incubation at 37 °C for 48 h. These halos were clearly distinguishable from the surrounding medium and were accompanied by fine granular precipitates in several isolates. The formation of precipitation zones indicated the ability of these isolates to deconjugate GDCA and supported their selection for further quantitative evaluation.

### 3.3. Semi-Quantitative Evaluation of Bile Salt Hydrolysis Percentage

To further compare the bile salt-hydrolyzing phenotype of the 105 BSH-positive isolates, semi-quantitative assays were performed. As shown in Table 1, considerable inter-strain variation was observed, with bile salt hydrolysis percentages ranging from 2.88% to 86.14%.

Among the tested isolates, C422 showed the highest hydrolysis percentage (86.14 ± 1.21%), followed by C415 (85.95 ± 1.33%), C410 (83.30 ± 1.89%), C427 (83.11 ± 1.47%), and C398 (82.93 ± 0.88%). In contrast, C411 (2.88 ± 0.06%), C329 (2.93 ± 0.17%), and C320 (3.78 ± 0.05%) exhibited very low bile salt hydrolysis capacity. Most isolates showed hydrolysis percentages between 10% and 30%, indicating moderate or low activity.

Based on these results, isolates with bile salt hydrolysis percentages greater than 55% were selected for subsequent cholesterol-removal assays. This threshold was used to retain strains with relatively high bile salt hydrolysis percentages for subsequent screening.

### 3.4. Preliminary In Vitro Cholesterol-Removal Phenotype of Selected Strains

The preliminary in vitro cholesterol-removal phenotype of 20 strains selected from the BSH screening was evaluated. As shown in Table 2, the cholesterol-removal rates varied markedly among strains, ranging from 14.48% to 67.79%.

C410 exhibited the highest cholesterol-removal rate (67.79 ± 2.23%), followed by C283 (63.29 ± 1.90%), C317 (63.09 ± 1.89%), C422 (62.86 ± 1.79%), and C289 (56.73 ± 2.74%). In contrast, C398 (14.48 ± 0.50%), C91 (22.92 ± 0.69%), and C392 (26.03 ± 0.78%) showed relatively weak cholesterol-removal ability.

Because cholesterol removal in vitro may involve multiple mechanisms, including bacterial assimilation, cell-surface adsorption, bile salt-mediated co-precipitation, and medium-related physicochemical changes, these results were interpreted only as a preliminary in vitro cholesterol-removal phenotype rather than evidence of cholesterol degradation or cholesterol-lowering capability. Fourteen strains with cholesterol-removal rates higher than 45% were selected for further physiological and biochemical characterization. This cut-off was used to prioritize strains with relatively high cholesterol-removal capacity while maintaining sufficient strain diversity for further evaluation. Representative images of the cholesterol-removal assay are provided in Supplementary Figure S1.

### 3.5. Physiological and Biochemical Characteristics of the Selected Strains

The growth characteristics of the selected strains under different temperature, NaCl, and pH conditions are summarized in Table 3. Most strains showed weak or no growth at 5 °C but were able to grow at 10 °C and 45 °C. Several strains, including C267, C277, C279, C283, C289, C410, C415, C422, and C423, maintained detectable growth at 50 °C, indicating relatively broad temperature tolerance.

Regarding salt tolerance, most isolates grew under 3.0% NaCl, whereas C40 showed markedly inhibited growth. C277, C279, C283, C289, C317, C410, C415, C422, and C423 also grew under 6.5% NaCl, suggesting stronger osmotic-stress tolerance.

For pH tolerance, most strains showed weak or no growth at pH 3.0–4.0, whereas growth improved under weakly acidic to near-neutral conditions. The selected strains also maintained growth under alkaline conditions, including pH 9.0 and pH 10.0. These results indicate that several strains possessed broad environmental adaptability, supporting their continued evaluation as candidate probiotics. Representative images of the physiological and biochemical characterization assays are provided in Supplementary Figure S2.

### 3.6. Cell-Surface Hydrophobicity and Auto-Aggregation as Adhesion-Related Surface Properties

**Figure 2:**
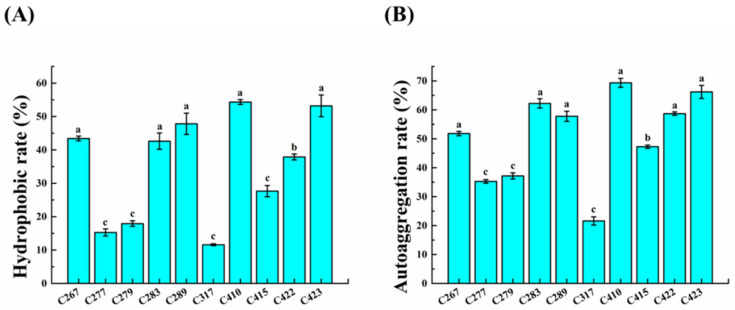
Cell-surface hydrophobicity and auto-aggregation ability as adhesion-related surface properties of LAB isolates. (**A**) Hydrophobicity ability (**B**) Auto-aggregation ability. Different lowercase letters indicate significant differences (*p* < 0.05).

Cell-surface hydrophobicity and auto-aggregation ability were evaluated as indirect in vitro indicators of adhesion-related surface properties. As shown in Figure 2, cell-surface hydrophobicity ranged from 11.60% to 54.35%, indicating substantial inter-strain variation.

C410 showed the highest hydrophobicity (54.35%), followed by C423 (53.20%) and C289 (52.30%). C317 showed the lowest hydrophobicity (11.60%), whereas C277 (15.00%) and C279 (18.00%) also exhibited relatively low values. C267 (43.00%), C283 (42.00%), and C422 (38.00%) showed moderate to high hydrophobicity.

Auto-aggregation rates ranged from 21.60% to 69.35%. C410 showed the highest auto-aggregation ability (69.35%), followed by C423 (66.22%), C283 (62.24%), and C289 (57.79%). The aggregation rates of C422 and C267 also exceeded 50%. Based on the combined criteria of hydrophobicity greater than 35% and auto-aggregation greater than 50%, six strains were selected for further in vitro functional evaluation.

### 3.7. Growth and Acid-Production Curves of Selected Strains

**Figure 3:**
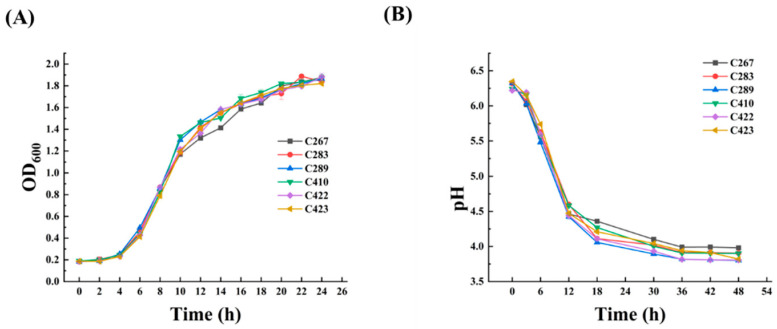
Growth and acid-production curves of six selected strains. (**A**) Growth curves. (**B**) Acid-production curves.

The growth and acid-production profiles of the six selected strains are shown in Figure 3. All strains entered the stationary phase after approximately 12 h of incubation. After 24 h, OD_600_ values exceeded 1.75 for all strains, with no significant differences among strains at the same time points (*p* > 0.05), indicating comparable growth performance under the tested culture conditions.

The pH of the fermentation broth decreased rapidly during the first 12 h and then gradually stabilized. After 48 h of incubation, the final pH values of all strains remained between 3.9 and 4.1. No significant differences were observed in acidification patterns among the strains (*p* > 0.05), suggesting similar acid-production dynamics during in vitro cultivation.

### 3.8. In Vitro Antioxidant Activity of the Selected Strains

**Figure 4:**
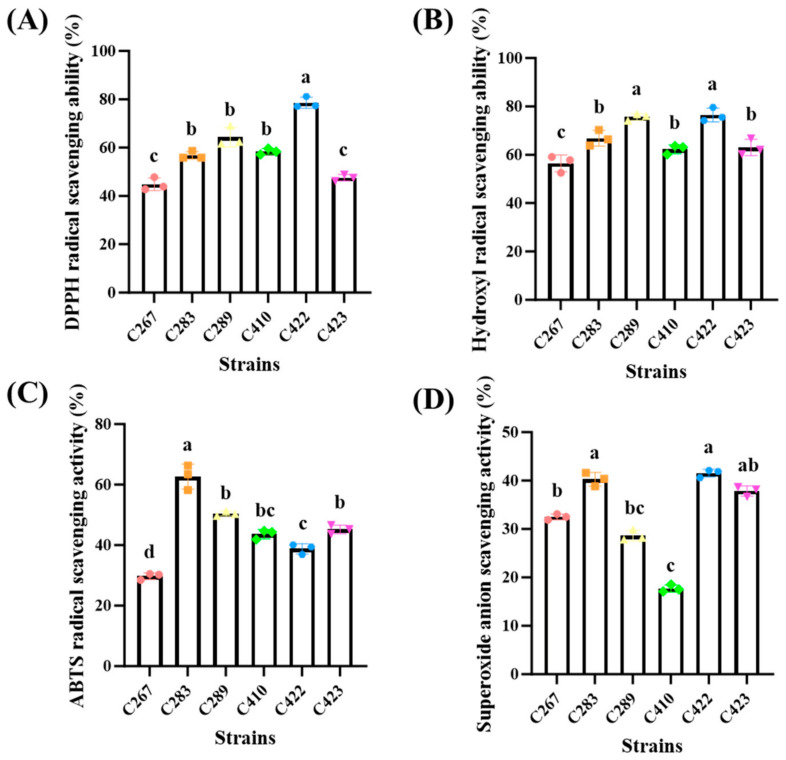
In vitro antioxidant activity of selected strains. (**A**) DPPH radical-scavenging rate. (**B**) Hydroxyl radical-scavenging rate. (**C**) ABTS radical-scavenging rate. (**D**) Superoxide anion-scavenging rate. Different lowercase letters indicate significant differences among strains within the same assay according to Tukey’s multiple-comparison test (*p* < 0.05).

The antioxidant activity of the selected strains was assessed using DPPH, hydroxyl radical, ABTS radical, and superoxide anion-scavenging assays (Figure 4A–D). All strains exhibited antioxidant activity to varying degrees, but the activity differed among strains and assay systems.

In the DPPH assay, C422 showed the highest scavenging activity, with a scavenging rate of 78.6%. C289, C410, and C283 showed moderate to high scavenging activities, ranging from 56.8% to 64.3%, whereas C267 and C423 showed relatively lower activity. In the hydroxyl radical-scavenging assay, C422 and C289 showed the highest activities, with scavenging rates of 76.4% and 75.6%, respectively. The remaining strains also showed scavenging rates above 60%, except C267, which showed the lowest activity at 56.4%.

In the ABTS assay, C283 exhibited the highest scavenging rate (62.6%), which was significantly higher than those of the other strains. C267 showed the lowest ABTS radical-scavenging activity (29.7%). For superoxide anion scavenging, C422 and C283 showed the highest activities, with scavenging rates of 41.5% and 40.3%, respectively, whereas C410 showed the lowest activity (17.7%).

Overall, the antioxidant activities of the selected strains varied depending on the assay system, suggesting strain-dependent differences in free radical-scavenging capacity. C422, C289, and C283 showed relatively consistent antioxidant performance across multiple assays, whereas the other strains exhibited more variable activities. These results provided additional functional information for the subsequent comprehensive selection of candidate strains.

### 3.9. Survival of Selected Strains Under Simulated Gastrointestinal Conditions

**Figure 5:**
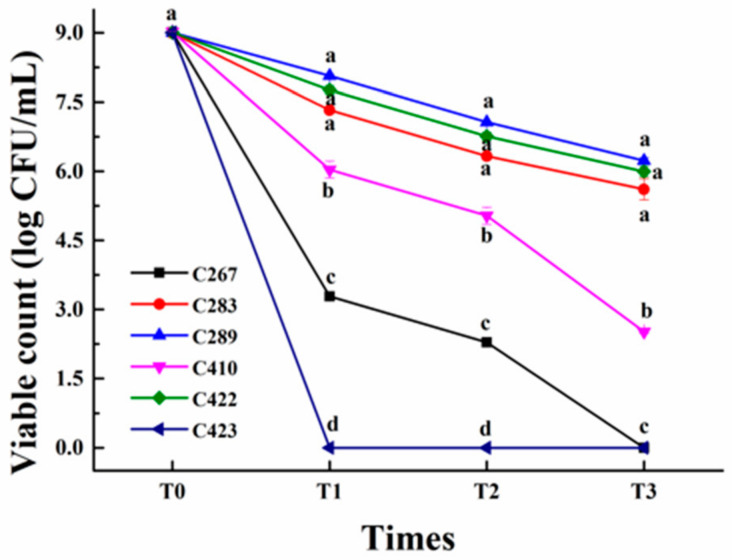
Survival of selected LAB strains under simulated gastrointestinal conditions. Viable counts are expressed as log CFU/mL. T0, initial count before simulated gastric fluid treatment; T1, count after simulated gastric fluid treatment; T2, count before simulated intestinal fluid treatment; T3, count after simulated intestinal fluid treatment. Different lowercase letters indicate significant differences (*p* < 0.05).

The survival of the six selected strains under simulated gastrointestinal conditions is shown in Figure 5. The initial viable counts of all strains were approximately 9.00 log CFU/mL, indicating comparable baseline cell densities.

After exposure to simulated gastric fluid, C289, C283, and C422 retained relatively high viable counts. At T1, the viable counts of C289, C283, and C422 were 8.07, 7.33, and 7.76 log CFU/mL, respectively. In contrast, C410 decreased to 6.04 log CFU/mL, while C267 and C423 were nearly undetectable. After subsequent simulated intestinal fluid treatment, C289, C283, and C422 continued to maintain higher viable counts than the other strains.

These results indicate that C289, C283, and C422 had better tolerance to simulated gastric and intestinal stresses than the other tested strains. Therefore, these three strains were selected as core candidates for taxonomic identification, safety evaluation, antibacterial testing, and organic acid analysis.

### 3.10. Phylogenetic Analysis of the Selected Strains

**Figure 6:**
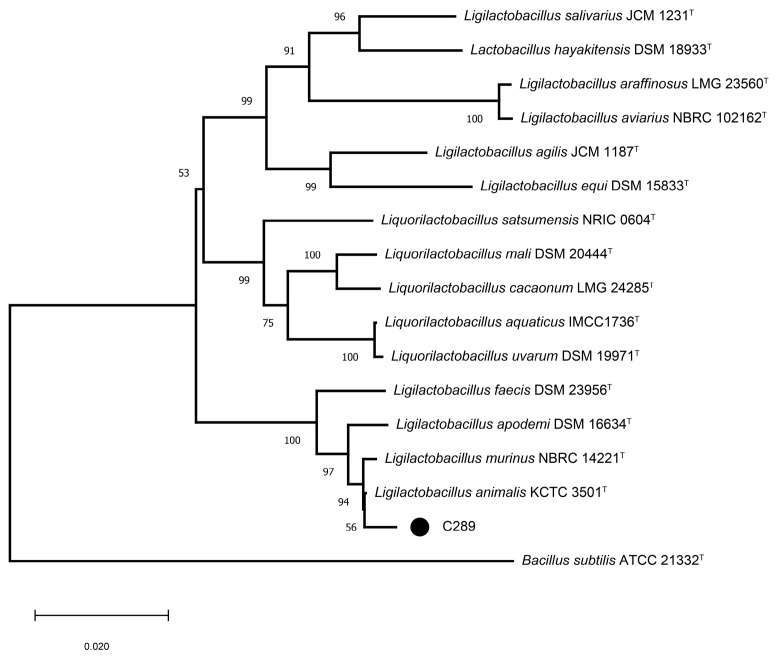
Phylogenetic tree showing the relative position of strain C289. The tree was constructed using the neighbor-joining method. *Bacillus subtilis* was used as the outgroup. Bootstrap values were obtained from 1000 replicates. The scale bar represents 1% sequence divergence.

**Figure 7:**
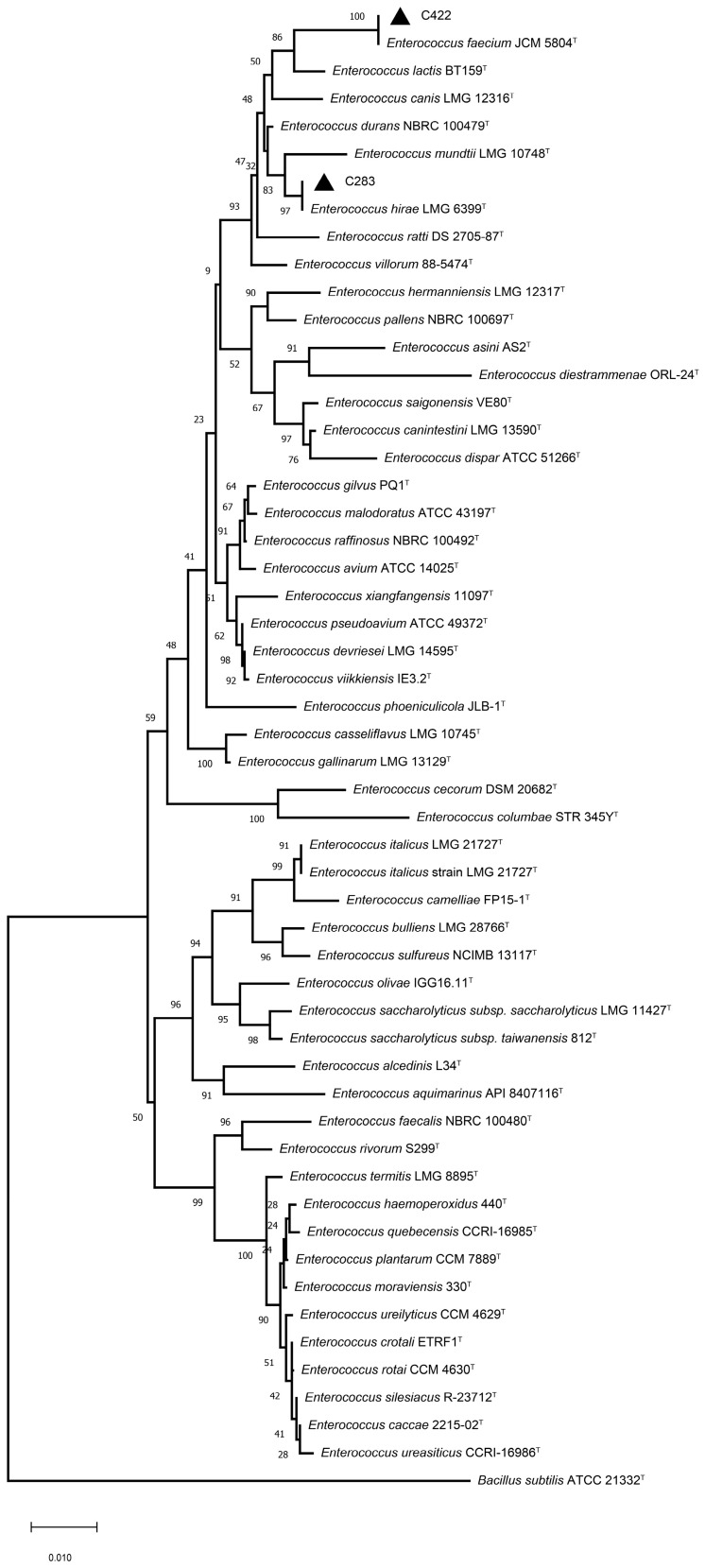
Phylogenetic tree showing the relative positions of strains C283 and C422. The tree was constructed using the neighbor-joining method. *Bacillus subtilis* was used as the outgroup. Bootstrap values were obtained from 1000 replicates. The scale bar represents 1% sequence divergence.

Phylogenetic analysis based on 16S rRNA gene sequences was performed to determine the taxonomic affiliation of the three core strains. As shown in Figure 6 and Figure 7, C289 clustered with *Ligilactobacillus animalis* KCTC 3501^T^, indicating that this strain belongs to *Ligilactobacillus animalis*.

C283 clustered with *Enterococcus hirae* LMG 6399^T,^ whereas C422 clustered with *Enterococcus faecium* JCM 5804^T^. These results provisionally identified the three core strains as *Ligilactobacillus animalis* C289, *Enterococcus hirae* C283, and *Enterococcus faecium* C422. Because 16S rRNA gene sequencing has limited resolution for some closely related species, further genome-level identification would strengthen the taxonomic assignment of these strains.

### 3.11. Preliminary Safety Evaluation of Selected Strains

#### 3.11.1. Hemolytic Activity

**Figure 8:**
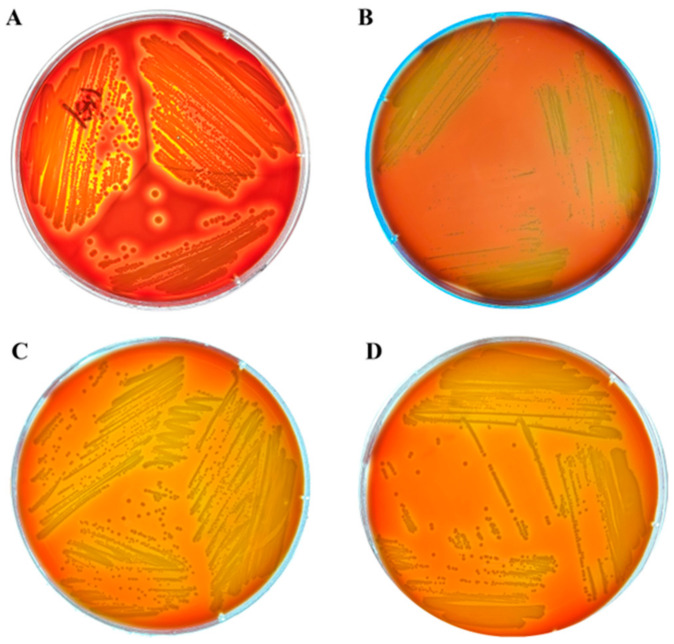
Hemolytic activity of selected LAB isolates. (**A**) Positive control, *Staphylococcus aureus* ATCC 29213^T^. (**B**) C283. (**C**) C289. (**D**) C422. The Chinese character “金” indicates Staphylococcus aureus, which was used as the positive control.

The hemolytic activity of the three core strains is shown in Figure 8. The positive control, *Staphylococcus aureus* ATCC 29213^T^, produced a clear transparent hemolytic zone on blood agar, indicating β-hemolytic activity. In contrast, no hemolytic zones were observed around colonies of C283, C289, or C422, and the surrounding blood agar remained intact.

These results indicate that the three tested strains showed no detectable hemolytic activity under the experimental conditions, supporting their preliminary safety profile.

#### 3.11.2. Antibiotic Susceptibility

The antibiotic susceptibility profiles of C283, C289, and C422 are shown in Table 4. C283 was resistant to ciprofloxacin and compound sulfamethoxazole; intermediate to gentamicin, ceftriaxone, tetracycline, and lincomycin; and sensitive to erythromycin, ampicillin, chloramphenicol, and penicillin. C289 was resistant to ciprofloxacin, compound sulfamethoxazole, and lincomycin; intermediate to gentamicin, ceftriaxone, erythromycin, ampicillin, and tetracycline; and sensitive to chloramphenicol and penicillin. C422 was resistant to gentamicin, ciprofloxacin, compound sulfamethoxazole, and lincomycin; intermediate to ceftriaxone, tetracycline, and chloramphenicol; and sensitive to erythromycin, ampicillin, and penicillin.

All three strains exhibited resistant or intermediate phenotypes to several antibiotics, indicating that their antibiotic susceptibility profiles should be interpreted with caution. These results provide preliminary phenotypic information but are insufficient to determine whether resistance determinants are intrinsic or transferable. Given that C283 and C422 belonged to the genus Enterococcus, further genome-based analysis of antibiotic resistance genes, virulence-associated genes, and mobile genetic elements is required before practical application. Representative images of the antibiotic susceptibility assay are provided in Supplementary Figure S3.

### 3.12. Antibacterial Activity of Selected Strains

The antibacterial activities of the three candidate strains against six indicator bacteria are shown in Table 5. C283 exhibited strong inhibitory activity against *Listeria monocytogenes*, *Bacillus subtilis*, and *Shigella dysenteriae*, moderate inhibitory activity against *Escherichia coli* and *Staphylococcus aureus*, and weak inhibitory activity against *Pseudomonas aeruginosa*.

C289 showed the broadest antibacterial spectrum among the three strains. It exhibited weak inhibitory activity against *P. aeruginosa*, moderate inhibitory activity against *S. aureus*, and strong inhibitory activity against the other four indicator bacteria, particularly *L. monocytogenes*. C422 showed pronounced inhibitory activity against *S. dysenteriae*, *L. monocytogenes*, and *E. coli*, and moderate inhibitory activity against the remaining indicator bacteria.

Overall, the three selected strains showed inhibitory activity against both Gram-positive and Gram-negative indicator bacteria under the tested conditions, although the inhibitory spectrum and intensity differed among strains. Representative agar plate images of the antibacterial activity assay are provided in Supplementary Figure S4.

### 3.13. Effects of pH Neutralization, Catalase, and Protease Treatments on Antibacterial Activity

As shown in Table 6, the fermentation liquids and cell-free supernatants of C283, C289, and C422 all exhibited antibacterial activity against *Escherichia coli* CICC 24189^T^. C289 and C422 showed stronger activity than C283, with inhibition zones ranging from 18.00 to 22.00 mm. After hydrogen peroxide-removal treatment, the antibacterial activity of the three strains was still maintained. In contrast, no inhibition zones were observed after treatment with proteinase K, pepsin, or trypsin. The antibacterial activity was strongly affected by pH. All three strains showed the strongest inhibition at pH 2.5, followed by gradually reduced activity at pH 3.5 and pH 4.5. No antibacterial activity was detected when the pH was adjusted to 5.5 or higher. Representative agar plate images showing the antibacterial activity after different treatments are provided in Supplementary Figure S5.

### 3.14. Organic Acid Contents in Fermentation Broths

**Figure 9:**
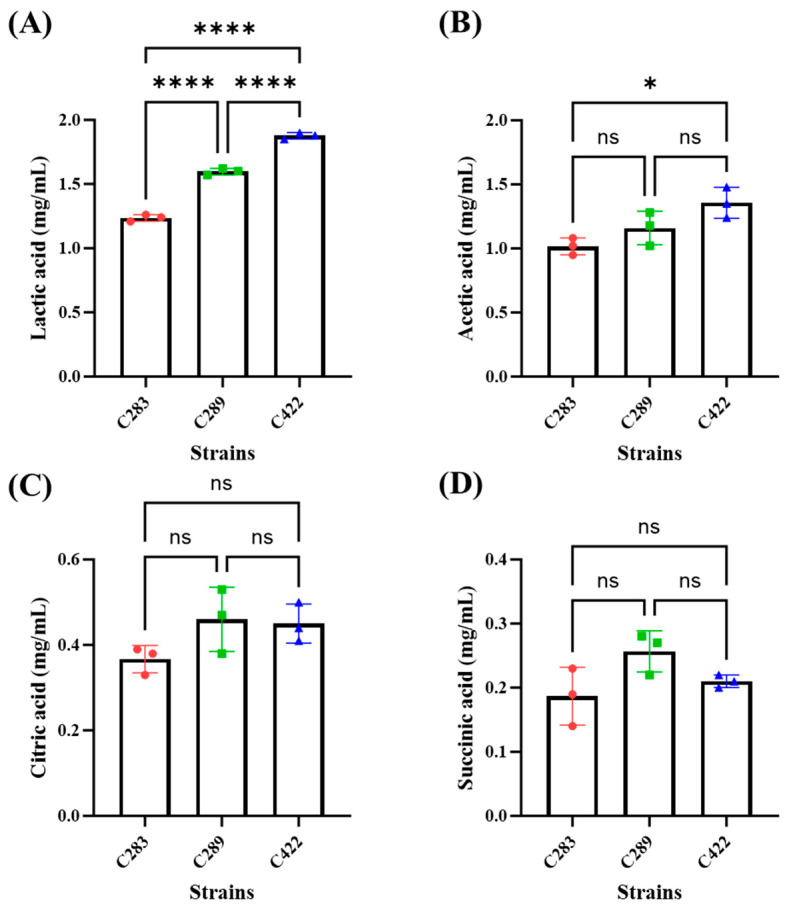
Organic acid contents in fermentation broths of selected strains. (**A**) Lactic acid content. (**B**) Acetic acid content. (**C**) Citric acid content. (**D**) Succinic acid content. * indicates a significant difference (*p* < 0.05); **** indicates a highly significant difference (*p* < 0.0001); ns indicates no significant difference (*p* > 0.05).

The organic acid profiles of the fermentation broths of the three candidate strains were analyzed using UPLC–Orbitrap–MS. As shown in Figure 9, lactic acid, acetic acid, citric acid, and succinic acid were detected as the major organic acids.

For lactic acid, significant differences were observed among strains (*p* < 0.0001). C422 produced the highest lactic acid concentration (1.88 mg/mL), indicating stronger lactic acid-producing capacity than the other two strains. For acetic acid, C422 also showed the highest concentration (1.36 mg/mL), followed by C289 (1.16 mg/mL) and C283 (1.02 mg/mL). The difference between C422 and C283 was statistically significant (*p* < 0.05). In contrast, citric acid and succinic acid concentrations differed only slightly among strains, with no significant differences observed (*p* > 0.05).

These results indicate that all three strains were capable of producing multiple organic acids, with lactic acid and acetic acid as the predominant metabolites. Among them, C422 showed the highest production of the two major organic acids, whereas C289 and C283 showed relatively moderate acid-production profiles.

## 4. Discussion

In the present study, approximately 700 lactic acid bacterial isolates were obtained from fecal samples collected from 300 healthy cats. Among the 200 isolates subjected to 16S rRNA gene sequencing, the predominant species were *Ligilactobacillus animalis* (34.5%) and *Ligilactobacillus salivarius* (19.0%), indicating that rod-shaped LAB, particularly *Ligilactobacillus* species, were predominant among the culturable LAB isolates obtained under the present isolation conditions. This finding is consistent with previous reports on the gut microbiota composition of healthy cats [34,35]. However, it should be noted that the taxonomic identification of the selected isolates was based on 16S rRNA gene sequencing, which provides useful preliminary identification but may not provide sufficient resolution for closely related species within the *Enterococcus* and *Ligilactobacillus* genera. Therefore, the species-level assignments of C283, C289, and C422 should be regarded as provisional. Future studies should confirm the taxonomic identities of these isolates using multilocus sequence analysis or whole-genome sequencing. Marker genes such as *sodA*, *tuf*, *groEL*, and *atpA* may improve species-level discrimination within *Enterococcus*, while *pheS*, *rpoA*, *groEL*, and *recA* may be useful for differentiating closely related *Ligilactobacillus* species. In addition, genome-based analyses, including average nucleotide identity, digital DNA–DNA hybridization, and core-genome phylogeny, are needed for definitive taxonomic confirmation.

Among the 700 isolates screened for bile salt hydrolase (BSH) activity, 105 were identified as BSH-positive. Further semi-quantitative analysis revealed that several strains, such as C422 (86.14%) and C415 (85.95%), exhibited relatively high bile salt hydrolysis percentages, markedly exceeding that of strains with moderate or low activity. BSH activity is commonly used as an in vitro screening trait for bacterial strains that may influence bile salt metabolism. Through the deconjugation of bile salts, BSH activity may alter bile acid solubility and intestinal reabsorption, thereby providing a possible basis for further studies on cholesterol metabolism [23,36]. From a theoretical perspective, BSH-mediated bile salt deconjugation may be relevant to lipid-related metabolism through several mechanisms. Conjugated bile acids are more water-soluble and are efficiently reabsorbed through enterohepatic circulation, whereas deconjugated bile acids generally have lower solubility and may be less efficiently reabsorbed in the intestine. This may increase fecal bile acid loss and stimulate hepatic conversion of cholesterol into bile acids to maintain the bile acid pool. In addition, bile acids are not only digestive molecules required for lipid emulsification and absorption, but also metabolic signaling molecules that may interact with receptors such as FXR and TGR5, which are involved in lipid, glucose, and energy metabolism. These pathways may be particularly relevant in cats because cats are obligate carnivores with high dietary fat utilization and a bile acid-dependent lipid digestion process. However, the present study did not analyze bile acid composition, fecal bile acid excretion, FXR/TGR5 signaling, serum lipid profiles, or host metabolic responses in cats. Therefore, the BSH activity observed in C283, C289, and C422 should be interpreted only as an in vitro bile acid-related screening phenotype, and its relevance to feline lipid metabolism requires further validation in feline cell models and in vivo feeding trials.

The present study also demonstrated marked differences in preliminary in vitro cholesterol-removal phenotypes among LAB strains. Strains such as C410, C283, and C317 showed cholesterol-removal rates above 60%, indicating relatively high cholesterol-removal rates under the tested culture conditions. However, cholesterol removal in vitro may involve multiple mechanisms, including bacterial assimilation, cell-surface adsorption, and bile salt-mediated co-precipitation, and therefore should be interpreted as a functional screening indicator rather than direct evidence of in vivo cholesterol-lowering efficacy. Recent studies have increasingly emphasized the critical role of BSH in cholesterol homeostasis. For example, Ertürkmen et al. (2023) reported that the expression levels of BSH genes, particularly *bsh3*, in *Lactiplantibacillus plantarum* and *Lacticaseibacillus paracasei* were closely associated with strain-specific cholesterol-removal capacity, providing a molecular basis for screening efficient cholesterol-lowering strains [37]. In addition, Zhao and colleagues constructed substrate-specific BSH mutant strains and demonstrated that BSH could influence intestinal FXR signaling by modulating the composition of the bile acid pool, thereby reducing host serum cholesterol levels in experimental models [38]. Collectively, the coexistence of high BSH activity and cholesterol-removal phenotype in strains such as C283, C317, and C410 is consistent with previous observations that these traits are frequently associated in probiotic candidate strains. These findings provided a basis for the subsequent multi-parameter screening of feline-derived LAB with relatively high BSH activity and in vitro cholesterol-removal capacity.

Physiological and biochemical characterization showed that C267, C277, C279, C283, C289, C317, C410, C415, C422, and C423 were able to grow under stressful conditions, including elevated temperature (50 °C), high salinity (6.5% NaCl), and strong alkalinity (pH 10.0). Such multi-stress tolerance suggests that these strains possess relatively broad environmental adaptability, which may be beneficial for strain handling, cultivation, and product development. However, tolerance to 50 °C does not directly indicate resistance to high-temperature pet food processing, and further processing-stability assays are required to evaluate their survival during practical manufacturing conditions. Previous studies have shown that long-term exposure to stressful environments can enhance the tolerance of lactic acid bacteria to acid, bile salts, and gastrointestinal fluids [39]. In addition, stress proteins such as DnaK and GroEL may be activated to maintain cellular homeostasis and improve survival under adverse conditions [40]. Therefore, the strains with superior stress tolerance identified in this study exhibit broad environmental adaptability and were considered suitable for further functional evaluation.

Cell-surface hydrophobicity and auto-aggregation ability were evaluated as indirect in vitro indicators of adhesion-related surface properties. Several strains showed favorable performance in both assays, with hydrophobicity values exceeding 50% and auto-aggregation rates exceeding 60%, suggesting potential adhesion-related advantages. These findings are consistent with previous reports that hydrophobicity and auto-aggregation may be associated with adhesion-related characteristics, although they cannot substitute for direct intestinal adhesion assays [41,42]. However, these parameters cannot fully represent actual intestinal adhesion or colonization ability in vivo. The mucus layer and glycoprotein composition of the feline intestine differ from those of humans and rodents [43]. Therefore, whether these feline-derived strains can adhere to or persist on the feline intestinal mucosa remains unknown and requires direct validation using feline mucus models, feline-derived intestinal epithelial cells, or other appropriate intestinal epithelial cell models. Host-derived bacteria may be better adapted to the gastrointestinal environment of their original host species. For instance, canine-derived *Enterococcus hirae* and *Enterococcus faecium* strains have been reported to adhere more strongly to canine intestinal mucosa than strains of the same species derived from chickens [44]. Similarly, canine-derived *Lactobacillus acidophilus* showed stronger adhesion to canine colonic mucus than porcine- or human-derived strains [45]. If intestinal persistence is demonstrated in future studies, it may help sustain bacterial activities such as bile salt deconjugation; however, this cannot be inferred from hydrophobicity and auto-aggregation assays alone. Therefore, the hydrophobicity and auto-aggregation properties of strains such as C283 and C422 should be interpreted only as adhesion-related surface characteristics and not as evidence of actual intestinal adhesion or colonization. Notably, hydrophobicity and auto-aggregation may also be associated with strain stability in functional products. For example, Shehata et al. found that LAB strains isolated from the honeybee stomach with strong hydrophobicity and auto-aggregation ability also survived well in fermented milk products, further indicating the relevance of these traits for functional product development [46]. Compared with these widely used strains, the isolates obtained in the present study were derived from healthy cats and therefore may have potential host-associated advantages in adapting to the feline gastrointestinal environment. However, the present study was based on in vitro screening only, and direct superiority over commercial probiotic strains cannot be concluded. In addition, comparisons across published studies are limited by differences in strain source, dosage, experimental design, target animals, and evaluation endpoints. Therefore, future feline feeding trials should include representative commercial probiotic strains as reference controls to directly compare gastrointestinal survival, safety, fecal characteristics, gut microbiota modulation, metabolic indicators, and host responses.

The antioxidant capacity of probiotic candidates is an important in vitro characteristic for protecting microbial cells against oxidative stress and may provide a basis for further evaluation of host oxidative status [47]. In the present study, strains C422, C289, and C283 exhibited antioxidant activity across multiple assay systems, including DPPH, hydroxyl radical, ABTS, and superoxide anion-scavenging assays. In particular, C422 showed DPPH and hydroxyl radical-scavenging rates of 78.6% and 76.4%, respectively, demonstrating strong in vitro antioxidant potential. Li et al. reported that antioxidant probiotics can enhance antioxidant enzyme activity and reduce intracellular hydrogen peroxide levels by up-regulating pathways such as Nrf2, thereby contributing to oxidative stress regulation [48]. Feline obesity is commonly accompanied by systemic oxidative imbalance and chronic low-grade inflammation [49]. Therefore, the antioxidant activity observed in strains such as C422 may be relevant to obesity-associated oxidative stress, although this potential effect remains to be confirmed in vivo. In addition, strong antioxidant capacity may improve the survival and functional stability of probiotic products during storage [46], which is of practical significance for the development of feline probiotic products.

The ability of probiotic candidates to survive harsh gastrointestinal conditions is an important prerequisite for further functional evaluation. In this study, the survival of six LAB strains in simulated gastrointestinal fluids was evaluated. The results showed that C289, C283, and C422 maintained relatively high viable counts after exposure to simulated gastric and intestinal fluids, indicating favorable acid and bile tolerance. It should be noted that the gastrointestinal physiology of cats differs from that of humans. Previous studies have reported that the gastric pH of healthy cats is approximately 2.7 under fasting conditions, with a range of 1.7–6.2, and approximately 2.0 after feeding, with a range of 1.1–3.3. The small intestinal pH is approximately 8.2 under fasting conditions, with a range of 7.6–8.7, and approximately 7.8 after feeding, with a range of 6.7–8.5 [50]. The pH values used in the present study, pH 2.5 for simulated gastric fluid and pH 8.0 for simulated intestinal fluid, therefore fall within the actual physiological pH range of the feline gastrointestinal tract. In addition, SGF exposure for 3 h and SIF exposure for 4 h are consistent with reported ranges for gastric emptying and small intestinal transit time in cats. Thus, the pH values and exposure durations used in this study reasonably reflect the stress conditions that probiotic candidate strains may encounter in the real feline gastrointestinal tract. Interestingly, these strains showed weak growth under acidic conditions at pH 3.0–4.0 in routine growth assays; however, under short-term simulated gastric stress at pH 2.5 for 3 h, C289, C283, and C422 still maintained high survival rates. This suggests that these strains possess stress-response capacity sufficient to support their passage through the feline stomach and entry into the intestine. Previous studies have shown that probiotics isolated from the target host intestine may exhibit better host adaptability and functional efficacy than strains derived from other hosts [51]. The relatively high survival of C289, C283, and C422 under the tested simulated gastrointestinal conditions supports their continued evaluation as feline-derived candidate strains. However, these results do not demonstrate intestinal persistence, colonization, or probiotic efficacy in cats.

Although strains such as C410, C317, and C415 showed strong BSH activity, cholesterol-removal capacity, or adhesion-related properties in earlier screening steps, their overall probiotic suitability was further evaluated using multiple functional criteria. In particular, survival under simulated gastrointestinal conditions was considered a critical selection factor, because probiotic candidates must remain viable during gastrointestinal transit to exert potential functional effects. Therefore, the superior gastrointestinal tolerance of C283, C289, and C422 contributed substantially to their final selection as core candidate strains.

Safety is a fundamental prerequisite for the application of probiotic candidates in food, feed, or veterinary contexts. Recent studies generally emphasize that only strains without toxicity and without transferable antibiotic resistance risks are suitable for further development as probiotic candidates [52]. In this study, C283, C289, and C422 did not produce hemolytic zones on blood agar, indicating that they lacked hemolytic activity and showed favorable preliminary safety characteristics for probiotic candidates. This result is consistent with the findings of Hussain et al., who reported that several non-hemolytic LAB strains isolated from traditional dairy products exhibited favorable probiotic potential [53]. Antibiotic susceptibility testing showed that all three strains remained sensitive to penicillin, an antibiotic of clinical relevance in feline medicine. Their susceptibility to ampicillin varied: C283 and C422 were sensitive, whereas C289 showed intermediate resistance. Zareie et al. also suggested that strains sensitive to commonly used antibiotics have greater application value when evaluating isolates from traditional cheese [54]. In addition, resistance of lactic acid bacteria to aminoglycosides is mainly attributed to the lack of cytochrome-mediated drug transport systems. This type of resistance is generally considered intrinsic, and the corresponding genes are usually located on the chromosome, with a low risk of horizontal transfer; therefore, it is not regarded as a major safety concern [55,56]. Although C283, C289, and C422 did not exhibit hemolytic activity, all three strains showed resistant or intermediate phenotypes to several antibiotics in the disk diffusion assay. Therefore, these results should be interpreted cautiously and regarded only as preliminary phenotypic safety information. Phenotypic antibiotic susceptibility testing alone cannot distinguish intrinsic resistance from acquired resistance, nor can it determine whether resistance determinants are located on mobile genetic elements such as plasmids, transposons, or integrons. This is particularly important for the two *Enterococcus* isolates, because some members of this genus may act as opportunistic pathogens and may harbor transferable antimicrobial resistance determinants. Therefore, the safety of C283, C289, and C422 cannot be confirmed based only on hemolysis testing and disk diffusion results. Future whole-genome sequencing should be performed to systematically analyze antimicrobial resistance genes, virulence-associated genes, plasmids, transposons, integrons, and other mobile genetic elements before these strains are considered for practical probiotic application.

Broad-spectrum antibacterial activity is an important functional attribute of probiotics. It not only contributes to inhibiting pathogenic bacterial growth and maintaining intestinal microbial balance, but may also help extend food shelf life and improve product quality. In the present study, the in vitro antibacterial activities of strains C283, C289, and C422 were evaluated against six common pathogenic or indicator bacteria. The results showed that all three strains exhibited broad-spectrum antibacterial properties to varying degrees. Among them, C289 displayed the strongest and broadest antibacterial spectrum, effectively inhibiting *Escherichia coli*, a common feline intestinal pathogen [57], as well as *Listeria monocytogenes*, *Bacillus subtilis*, and *Shigella dysenteriae*. C283 and C422 also showed strong inhibitory activity against specific bacteria, such as *B. subtilis* and *S. dysenteriae*. From the perspective of feline health, the inhibitory effects of strains such as C289 against common intestinal pathogens, including *E. coli*, suggest that these isolates may contribute to suppressing potentially harmful intestinal bacteria [57]. Moreover, feline obesity is often accompanied by gut microbial dysbiosis and an increased abundance of potentially pathogenic bacteria [58]. The antibacterial activity observed under the tested in vitro conditions provides additional functional characterization of the selected isolates. However, its relevance to intestinal microbial composition, obesity-associated dysbiosis, or body-weight regulation in cats remains unknown and requires in vivo investigation.

LAB can produce multiple antimicrobial metabolites, including organic acids, hydrogen peroxide, and bacteriocin-like proteinaceous compounds; therefore, inhibition zones in agar diffusion assays should not be directly attributed to a single antimicrobial substance [59]. In the present study, the antibacterial activity of C283, C289, and C422 against *Escherichia coli* CICC 24189^T^ was strongly pH-dependent, as the activity was maintained under acidic conditions but disappeared when the pH was adjusted to 5.5 or above. This result is consistent with previous findings that organic acids can inhibit *E. coli* by damaging the cell barrier, increasing membrane permeability, and affecting biofilm formation [60]. Catalase treatment did not reduce the antibacterial activity, suggesting that H_2_O_2_ was not the main inhibitory factor. Similar control strategies using pH adjustment, catalase treatment, and proteolytic enzymatic treatment have also been used to distinguish the antimicrobial contributors in LAB cell-free supernatants [61]. Taken together, the antibacterial activity of these strains was mainly associated with organic acid-mediated acidification rather than H_2_O_2_ or bacteriocin-like proteinaceous compounds under the present experimental conditions.

Organic acids are important fermentation metabolites of lactic acid bacteria. They can regulate the pH of the local environment, inhibit undesirable bacteria, and participate in host–microbe metabolic interactions. In this study, UPLC–Orbitrap–MS analysis showed that lactic acid, acetic acid, citric acid, and succinic acid were detected in the fermentation broths of C283, C289, and C422, indicating active organic acid metabolism during fermentation. Lactic acid and acetic acid were the predominant metabolites. Among them, acetate is a short-chain fatty acid involved in energy metabolism and host–microbe signaling. Previous studies have shown that short-chain fatty acids can regulate energy homeostasis and lipid metabolism through GPR41 and GPR43 signaling pathways [62,63]. In cats, microbiota-derived short-chain fatty acids have been associated with improved intestinal barrier function and reduced systemic inflammation [64,65]. Therefore, the organic acid profiles of these strains may partly contribute to their observed antibacterial activity and may provide useful metabolic information for future studies. The absence of detectable butyrate should also be noted. Butyrate is widely regarded as an important microbial metabolite associated with intestinal epithelial energy supply, tight-junction integrity, and gut barrier health. Therefore, the present results indicate that C283, C289, and C422 were not direct butyrate-producing strains under the tested in vitro fermentation conditions. Accordingly, no conclusion can be drawn regarding butyrate-mediated barrier protection based on the current data. However, lactic acid and acetic acid produced by LAB may potentially serve as substrates for cross-feeding by intestinal butyrate-producing bacteria in vivo. Whether these strains can indirectly promote butyrate production within the feline gut microbiota requires further investigation using microbiota-based fermentation systems or feline feeding trials.

Several limitations of this study should be acknowledged. First, all evaluations were conducted under in vitro conditions and therefore cannot fully reproduce the complex gastrointestinal environment, microbial interactions, and host responses in cats. Second, although BSH activity and preliminary in vitro cholesterol-removal phenotypes were used as key screening criteria, the molecular mechanisms underlying these phenotypes were not investigated. In particular, heat-inactivated bacterial controls, no-bile-salt controls, and biomass-normalized calculations were not included in the cholesterol-removal assay; therefore, the relative contributions of adsorption, assimilation, bile salt-mediated co-precipitation, medium-related changes, and bacterial biomass could not be determined. Third, the present in vitro assays did not evaluate actual intestinal adhesion or colonization using Caco-2, HT-29, IPEC-J2, feline-derived intestinal epithelial cells, feline mucus models, or feline in vivo models. In addition, blood lipid regulation, changes in lipid metabolism, body-weight reduction, or improvement in obesity-associated metabolic abnormalities in cats were not assessed. Finally, because two of the selected strains belong to the genus *Enterococcus*, whole-genome sequencing will be necessary to assess potential virulence determinants, transferable antimicrobial resistance genes, and other safety-related genetic elements before practical application.

## 5. Conclusions

In conclusion, the present study applied a stepwise in vitro screening strategy to feline-derived LAB and identified three candidate isolates: C283, provisionally identified as *Enterococcus hirae*; C289, provisionally identified as *Ligilactobacillus animalis*; and C422, provisionally identified as *Enterococcus faecium*. Under the tested in vitro conditions, these isolates exhibited BSH activity, cholesterol-removal capacity, tolerance to simulated gastrointestinal conditions, adhesion-related surface properties, antioxidant activity, antibacterial activity, and organic acid production. No detectable hemolytic activity was observed; however, all three isolates exhibited resistant or intermediate phenotypes to multiple antibiotics. The present findings support the further investigation of these isolates but do not demonstrate intestinal colonization, host-specific adaptation, regulation of blood lipids or lipid metabolism, body-weight reduction, or improvement in obesity-associated metabolic abnormalities in cats. Further studies should include genome-based safety assessment, particularly for the two *Enterococcus* strains, mechanistic investigations, and appropriately designed in vivo experiments before these isolates can be considered for practical probiotic application.

## Figures and Tables

**Table 1:** Bile salt hydrolysis percentages of LAB isolates.

Isolate	Bile Salt Hydrolysis Percentage (%)	Isolate	Bile Salt Hydrolysis Percentage (%)	Isolate	Bile Salt Hydrolysis Percentage (%)
C27	16.96 ± 0.24	C266	15.95 ± 0.18	C394	14.10 ± 0.52
C30	17.54 ± 0.26	C267	80.37 ± 1.73	C395	15.06 ± 0.67
C31	55.48 ± 0.51	C268	12.94 ± 0.38	C397	7.35 ± 0.31
C32	14.96 ± 0.13	C269	19.65 ± 0.26	C398	82.93 ± 0.88
C33	17.30 ± 0.25	C270	9.60 ± 0.18	C399	8.30 ± 0.14
C34	5.17 ± 0.31	C273	13.14 ± 0.15	C400	17.89 ± 0.32
C35	16.38 ± 0.17	C275	13.39 ± 0.41	C401	35.29 ± 0.51
C36	5.90 ± 0.22	C276	5.68 ± 0.34	C402	26.54 ± 0.19
C38	15.68 ± 0.12	C277	69.04 ± 0.97	C403	13.79 ± 0.05
C39	18.54 ± 0.26	C278	6.44 ± 0.74	C404	24.03 ± 0.47
C40	75.81 ± 0.91	C279	67.70 ± 1.73	C405	3.93 ± 0.11
C41	18.40 ± 0.27	C280	12.14 ± 0.25	C406	44.39 ± 0.42
C42	13.66 ± 0.08	C281	10.99 ± 0.33	C407	4.29 ± 0.16
C43	8.93 ± 0.18	C282	15.03 ± 0.42	C408	34.70 ± 0.33
C44	16.86 ± 0.24	C283	75.45 ± 1.18	C409	6.36 ± 0.69
C45	71.81 ± 1.45	C284	13.12 ± 0.38	C410	83.30 ± 1.89
C50	11.46 ± 0.24	C285	8.01 ± 0.09	C411	2.88 ± 0.06
C52	16.73 ± 0.14	C287	7.53 ± 0.21	C413	34.58 ± 0.30
C72	5.96 ± 0.12	C289	80.38 ± 0.86	C414	4.46 ± 0.09
C86	29.42 ± 0.39	C317	76.33 ± 1.22	C415	85.95 ± 1.33
C87	9.76 ± 0.25	C320	3.78 ± 0.05	C416	5.69 ± 0.23
C88	10.39 ± 0.21	C329	2.93 ± 0.17	C417	17.59 ± 0.35
C89	17.71 ± 0.26	C332	10.79 ± 0.26	C418	8.03 ± 0.17
C91	73.42 ± 1.87	C334	9.35 ± 0.24	C419	18.24 ± 0.37
C92	24.67 ± 0.10	C335	3.97 ± 0.06	C420	7.75 ± 0.17
C93	23.32 ± 0.17	C339	4.92 ± 0.12	C421	10.20 ± 0.21
C94	5.67 ± 0.23	C340	58.25 ± 0.74	C422	86.14 ± 1.21
C98	25.20 ± 0.11	C341	5.75 ± 0.23	C423	81.19 ± 0.84
C184	5.03 ± 0.21	C360	13.86 ± 0.74	C424	4.40 ± 0.09
C205	17.43 ± 0.15	C361	13.52 ± 0.29	C425	44.54 ± 0.93
C214	13.69 ± 0.24	C382	15.02 ± 0.16	C426	76.58 ± 2.54
C215	74.02 ± 0.86	C386	4.78 ± 0.09	C427	83.11 ± 1.47
C231	14.23 ± 0.29	C389	4.73 ± 0.21	C462	14.36 ± 0.29
C237	14.86 ± 0.25	C390	5.03 ± 0.34	C463	3.60 ± 0.08
C250	10.50 ± 0.19	C392	65.82 ± 0.98	C475	21.79 ± 0.75

**Table 2:** Cholesterol-removal rates of selected LAB isolates.

Isolate	Cholesterol-Removal Rates (%)	Isolates	Cholesterol-Removal Rates (%)
C31	33.12 ± 0.99	C317	63.09 ± 1.89
C40	48.42 ± 1.45	C340	39.94 ± 1.20
C45	45.28 ± 1.36	C392	26.03 ± 0.78
C91	22.92 ± 0.69	C398	14.48 ± 0.50
C215	43.91 ± 1.23	C410	67.79 ± 2.23
C267	51.49 ± 1.54	C415	45.13 ± 1.35
C277	49.44 ± 1.73	C422	62.86 ± 1.79
C279	54.81 ± 1.64	C423	53.94 ± 1.62
C283	63.29 ± 1.90	C426	50.79 ± 0.52
C289	56.73 ± 2.74	C427	46.63 ± 1.40

**Table 3:** Growth of selected LAB isolates under different temperature, NaCl, and pH conditions.

Isolates	Temperature (°C)				NaCl (*w*/*v*, %)		pH								
	5	10	45	50	3.0	6.5	3.0	3.5	4.0	4.5	5.0	5.5	6.0	9.0	10.0
C40	−	w	+	w	w	−	−	−	w	+	+	+	+	+	+
C45	−	w	+	w	+	−	−	−	w	+	+	+	+	+	+
C267	−	+	+	+	+	w	−	+	+	+	+	+	+	+	+
C277	−	+	+	+	+	+	−	+	+	+	+	+	+	+	+
C279	−	+	+	+	+	+	w	+	+	+	+	+	+	+	+
C283	w	+	+	+	+	+	−	+	+	+	+	+	+	+	+
C289	w	+	+	+	+	+	−	+	+	+	+	+	+	+	+
C317	w	+	+	w	+	+	w	+	+	+	+	+	+	+	+
C410	w	+	+	+	+	+	−	+	+	+	+	+	+	+	+
C415	w	+	+	+	+	+	−	+	+	+	+	+	+	+	+
C422	−	+	+	+	+	+	w	+	+	+	+	+	+	+	+
C423	−	+	+	+	+	+	−	+	+	+	+	+	+	+	+
C426	−	+	+	w	−	−	−	−	−	−	+	+	+	+	+
C427	−	w	+	w	w	w	−	−	−	w	+	+	+	+	+

+, Normal growth; w, weak growth; −, no growth.

**Table 4:** Antibiotic susceptibility of selected strains.

Isolate	GEN	CIP	CTR	E	AMP	TET	SXT	C	MY	PEN
C283	I	R	I	S	S	I	R	S	I	S
C289	I	R	I	I	I	I	R	S	R	S
C422	R	R	I	S	S	I	R	I	R	S

S, sensitive; I, intermediate; R, resistant. Antibiotic concentrations are expressed as µg/disk: gentamicin, GEN, 10; ciprofloxacin, CIP, 5; ceftriaxone, CTR, 30; erythromycin, E, 15; ampicillin, AMP, 10; tetracycline, TET, 30; compound sulfamethoxazole, SXT, 25; chloramphenicol, C, 30; lincomycin, MY, 2; and penicillin, PEN, 10.

**Table 5:** Antibacterial spectrum of selected strains.

Isolates	Indicator Bacteria					
	*P. aeruginosa*	*S. aureus*	*L. monocytogenes*	*E. coli*	*B. subtilis*	*S. dysenteria*
C283	+	++	+++	++	+++	++++
C289	+	++	++++	+++	+++	+++
C422	++	++	+++	+++	++	++++

+, inhibition-zone diameter of 10.00–14.00 mm; ++, inhibition-zone diameter of 14.00–18.00 mm; +++, inhibition-zone diameter of 18.00–22.00 mm; ++++, inhibition-zone diameter greater than 22.00 mm. The inhibition-zone diameter includes the diameter of the well punch, 10.00 mm. *P. aeruginosa*, *Pseudomonas aeruginosa* CICC 23694^T^; *S. aureus*, *Staphylococcus aureus* ATCC 29213^T^; *L. monocytogenes*, *Listeria monocytogenes* CICC 23929^T^; *E. coli*, *Escherichia coli* CICC 24189^T^; *B. subtilis*, *Bacillus subtilis* CICC 10275^T^; *S. dysenteriae*, *Shigella dysenteriae* CICC 23829^T^.

**Table 6:** Antimicrobial activity of C283, C289 and C422 to EPEC after different treatments.

Treatment	C283 Antimicrobial Activity	C289 Antimicrobial Activity	C422 Antimicrobial Activity
Fermentation liquid	++	+++	+++
Supernatant	++	+++	+++
Hydrogen peroxide	+++	+++	+++
Proteinase K	−	−	−
Pepsinum	−	−	−
Trypsase	−	−	−
2.5	++++	++++	++++
3.5	+++	+++	+++
4.5	++	++	++
5.5	−	−	−
6.5	−	−	−
7	−	−	−
10	−	−	−

Note: ++, diameter of the inhibition zone: 14.00–18.00 mm; +++, 18.00–22.00 mm; ++++, more than 22.00 mm, −, no inhibition zone was detected; the diameter of the inhibition zone included that of the hole puncher (10.00 mm).

## Data Availability

The data generated in this study are presented and discussed in the manuscript. The data generated in this study are presented and discussed in the manuscript. The 16S rRNA gene sequences of strains C283, C289, and C422 have been deposited in the GenBank repository under accession numbers PZ572959, PZ572957, and PZ572958, respectively.

## Supplementary Materials

The following supporting information can be downloaded at: https://www.mdpi.com/article/10.3390/microorganisms14071466/s1, Figure S1: Representative images of the cholesterol-removal assay corresponding to Table 2; Figure S2: Representative images of the physiological and biochemical characterization assays corresponding to Table 3; Figure S3: Representative images of the antibiotic susceptibility assay corresponding to Table 4; Figure S4: Representative agar plate images of the antibacterial activity assay corresponding to Table 5; Figure S5: Representative agar plate images showing antibacterial activity after different treatments corresponding to Table 6.

## Abbreviations

The following abbreviations are used in this manuscript:

LAB	Lactic Acid Bacteria
BSH	Bile Salt Hydrolase
SGF	Simulated Gastric Fluid
SIF	Simulated Intestinal Fluid
OD	Optical Density
PCR	Polymerase Chain Reaction
rRNA	Ribosomal RNA
DPPH	2,2-Diphenyl-1-Picrylhydrazyl
ABTS	2,2′-Azino-bis(3-Ethylbenzothiazoline-6-Sulfonic Acid)
ROS	Reactive Oxygen Species
SCFA	Short-Chain Fatty Acid
FXR	Farnesoid X Receptor
MS	Mass Spectrometry
GDCA	Glycodeoxycholic Acid
PBS	Phosphate-Buffered Saline
MRS	de Man–Rogosa–Sharpe
AMPK	AMP-activated protein kinase
SREBP-1c	Sterol Regulatory Element-Binding Protein-1c
FAS	Fatty Acid Synthase
SD	Standard Ddeviation
3NPH	3-Nitrophenylhydrazine
UPLC	Ultra-Performance Liquid Chromatography
BLAST	Basic Local Alignment Search Tool
TGR5	Takeda G protein-coupled Receptor 5

## References

[1] Öhlund M., Palmgren M., Holst B.S. (2018). Overweight in adult cats: A cross-sectional study. Acta Vet. Scand..

[2] Godfrey H., Morrow S., Abood S.K., Verbrugghe A. (2024). Identifying the target population and preventive strategies to combat feline obesity. J. Feline Med. Surg..

[3] Hanford R., Linder D.E. (2021). Impact of obesity on quality of life and owner ‘s perception of weight loss programs in cats. Vet. Sci..

[4] Clark M., Hoenig M. (2021). Feline comorbidities: Pathophysiology and management of the obese diabetic cat. J. Feline Med. Surg..

[5] Martins T.D.O., Ramos R.C., Possidonio G., Bosculo M.R.M., Oliveira P.L., Costa L.R., Zamboni V.A.G., Marques M.G., De Almeida B.F.M. (2023). Feline obesity causes hematological and biochemical changes and oxidative stress—A pilot study. Vet. Res. Commun..

[6] Ma X., Brinker E., Graff E.C., Cao W., Gross A.L., Johnson A.K., Zhang C., Martin D.R., Wang X. (2022). Whole-genome shotgun metagenomic sequencing reveals distinct gut microbiome signatures of obese cats. Microbiol. Spectr..

[7] Opetz D.L., Oba P.M., Lin C.Y., Ren P., Swanson K.S. (2024). Restricted feeding of weight control diets induces weight loss and affects body composition, voluntary physical activity, blood metabolites, hormones, and oxidative stress markers, and fecal metabolites and microbiota of obese cats. J. Anim. Sci..

[8] Wall M., Cave N.J., Vallee E. (2019). Owner and cat-related risk factors for feline overweight or obesity. Front. Vet. Sci..

[9] Antakyalioglu B., Ozturan Y.A., Parlatir Y., Akin I., Ural K. (2025). Intragastric botulinum toxin-A injection: A novel approach to successfully manage feline obesity as an alternative technique to conventional treatment. Vet. Rec. Case Rep..

[10] Zavišić G., Ristić S., Rikalović M., Petković B., Janković D., Vukadinović A., Petričević S. (2022). Beneficial effects of probiotic supplementation on glucose and triglycerides in a mouse model of metabolic syndrome. J. Funct. Foods.

[11] Qian Y., Li M., Wang W., Wang H., Zhang Y., Hu Q., Zhao X., Suo H. (2019). Effects of *Lactobacillus casei* YBJ02 on lipid metabolism in hyperlipidemic mice. J. Food Sci..

[12] Lacroix S., Leblanc N., Abolghasemi A., Paris-Robidas S., Martin C., Frappier M., Flamand N., Silvestri C., Raymond F., Millette M. (2023). Probiotic interventions promote metabolic health in high fat-fed hamsters in association with gut microbiota and endocannabinoidome alterations. Benef. Microbes.

[13] Zhang Y., Yao D., Huang H., Zhang M., Sun L., Su L., Zhao L., Guo Y., Jin Y. (2023). Probiotics increase intramuscular fat and improve the composition of fatty acids in Sunit sheep through the adenosine 5′-monophosphate-activated protein kinase (AMPK) signaling pathway. Food Sci. Anim. Resour..

[14] Agolino G., Pino A., Vaccalluzzo A., Cristofolini M., Solieri L., Caggia C., Randazzo C.L. (2024). Bile salt hydrolase: The complexity behind its mechanism in relation to lowering-cholesterol lactobacilli probiotics. J. Funct. Foods.

[15] Su Y., Ren J., Zhang J., Zheng J., Zhang Q., Tian Y., Zhang Y., Jiang Y., Zhang W. (2024). *Lactobacillus paracasei* JY062 alleviates glucolipid metabolism disorders via the adipoinsular axis and gut microbiota. Nutrients.

[16] Sun J., Gu X., Zhang H., Zhao L., Wang J., Wang X., Tao H., Wang Z., Han B. (2025). Application of probiotics in cats and dogs: Benefits and mechanisms. Vet. Sci..

[17] Klinmalai P., Kamonpatana P., Sodsai J., Srisa A., Promhuad K., Laorenza Y., Kovitvadhi A., Areerat S., Seubsai A., Nakphaichit M. (2025). Probiotics in pet food: A decade of research, patents, and market trends. Foods.

[18] Zha M., Zhu S., Chen Y. (2024). Probiotics and cat health: A review of progress and prospects. Microorganisms.

[19] He W., Connolly E.D., Wu G. (2024). Characteristics of the digestive tract of dogs and cats. Adv. Exp. Med. Biol..

[20] Holzapfel W.H., Todorov S.D. (2023). Special issue: Beneficial properties and safety of lactic acid bacteria. Microorganisms.

[21] Wang W., Dong H., Chen Q., Chang X., Wang L., Miao C., Chen S., Chen L., Wang R., Ge S. (2024). Antibacterial Efficacy of Feline-Derived Lactic Acid Bacteria against Enteropathogenic *Escherichia coli*: A Comprehensive In Vitro Analysis. Fermentation.

[22] Dong L., Li Y. (2022). Survival during simulated digestion, bacteriocin production, and bile salt hydrolase activities of lactic acid bacteria in poi, a fermented Hawaiian food. Curr. Dev. Nutr..

[23] Guo C.F., Zhang L.W., Li J.Y., Zhang Y.C., Xue C.H., Yi H.X., Du M., Han X. (2012). Screening of bile salt hydrolase-active lactic acid bacteria for potential cholesterol-lowering probiotic use. Adv. Mater. Res..

[24] Chabi I.B., Akogou F.U.G., Zannou O., Atchadé J.A., Adéyèmi D.A., Alamri A.S., Galanakis C.M., Kayodé A.P.P. (2024). Lactic acid bacteria from a traditional starter (kpètè-kpètè) of Benin opaque sorghum beer: Probiotic characteristics, cholesterol-lowering capacity, and exopolysaccharides production. Biomass Convers. Biorefinery.

[25] Zhang M., Wang X., Cui M., Wang Y., Jiao Z., Tan Z. (2018). Ensilage of oats and wheatgrass under natural alpine climatic conditions by indigenous lactic acid bacteria species isolated from high-cold areas. PLoS ONE.

[26] Wang W., Ma H., Yu H., Qin G., Tan Z., Wang Y., Pang H. (2020). Screening of *Lactobacillus plantarum subsp. plantarum* with potential probiotic activities for inhibiting ETEC K88 in weaned piglets. Molecules.

[27] Düz M., Doğan Y.N., Doğan O. (2020). Antioxidant activity of *Lactobacillus plantarum*, *Lactobacillus sake* and *Lactobacillus curvatus strains* isolated from fermented Turkish sucuk. An. Acad. Bras. Cienc..

[28] Sanna D., Fadda A. (2022). Role of the hydroxyl radical-generating system in the estimation of the antioxidant activity of plant extracts by electron paramagnetic resonance (EPR). Molecules.

[29] Wu D., Li H., Wang X., Chen R., Gong D., Long D., Huang X., Tang Z., Zhang Y. (2025). Screening and whole-genome analysis of probiotic lactic acid bacteria with potential antioxidants from yak milk and dairy products in the Qinghai—Tibet Plateau. Antioxidants.

[30] Zhang B., Wang Y., Tan Z., Li Z., Jiao Z., Huang Q. (2016). Screening of probiotic activities of *lactobacilli* strains isolated from traditional Tibetan Qula, a raw yak milk cheese. Anim. Biosci..

[31] Niu K.M., Kothari D., Cho S.B., Han S.G., Song I.G., Kim S.C., Kim S.K. (2019). Exploring the probiotic and compound feed fermentative applications of *Lactobacillus plantarum* SK1305 isolated from Korean green chili pickled pepper. Probiotics Antimicrob. Proteins.

[32] de Souza B.M.S., Borgonovi T.F., Casarotti S.N., Todorov S.D., Penna A.L.B. (2019). *Lactobacillus casei* and *Lactobacillus fermentum* strains isolated from mozzarella cheese: Probiotic potential, safety, acidifying kinetic parameters and viability under gastrointestinal tract conditions. Probiotics Antimicrob. Proteins.

[33] Sirichokchatchawan W., Pupa P., Praechansri P., Am-In N., Tanasupawat S., Sonthayanon P., Prapasarakul N. (2018). Autochthonous lactic acid bacteria isolated from pig faeces in Thailand show probiotic properties and antibacterial activity against enteric pathogenic bacteria. Microb. Pathog..

[34] Li Z., Di D., Sun Q., Yao X., Wei J., Li B., Liu K., Shao D., Qiu Y., Liu H. (2022). Comparative analyses of the gut microbiota in growing Ragdoll cats and Felinae cats. Animals.

[35] Masuoka H., Shimada K., Kiyosue-Yasuda T., Kiyosue M., Oishi Y., Kimura S., Ohashi Y., Fujisawa T., Hotta K., Yamada A. (2017). Transition of the intestinal microbiota of cats with age. PLoS ONE.

[36] Tsai C., Lin P., Hsieh Y., Zhang Z., Wu H., Huang C. (2014). Cholesterol-lowering potentials of lactic acid bacteria based on bile-salt hydrolase activity and effect of potent strains on cholesterol metabolism in vitro and in vivo. Sci. World J..

[37] Ertürkmen P., Fırıncıoğulları B., Öner Z. (2023). The expression levels of genes responsible for the enzymatic activity of bile salt hydrolase (BSH) and the relationship of cholesterol assimilation in *L. plantarum* and *L. paracasei*. Curr. Microbiol..

[38] Zhao M., Kuang W., Yang J., Liu Y., Yang M., Chen Y., Zhu H., Yang Y. (2024). Cholesterol lowering in diet-induced hypercholesterolemic mice using *Lactobacillus* bile salt hydrolases with different substrate specificities. Food Funct..

[39] Ming H., Xu D., Guo Z., Liu Y. (2016). Adaptive evolution of *Lactobacillus casei* under acidic conditions enhances multiple-stress tolerance. Food Sci. Technol. Res..

[40] Chen M., Tang H., Chiang M. (2017). Effects of heat, cold, acid and bile salt adaptations on the stress tolerance and protein expression of kefir-isolated probiotic *Lactobacillus kefiranofaciens* M1. Food Microbiol..

[41] Narayanan R., Keerthi T.R.K. (2024). In vitro analysis on the adhesion potential of *Lactiplantibacillus plantarum* from infant faeces and its gastrointestinal localization, growth promotion, and immunomodulation in Wistar rats: A preliminary study. Lett. Appl. Microbiol..

[42] Margalho L.P., Jorge G.P., Noleto D.A., Silva C.E., Abreu J.S., Piran M.V., Brocchi M., Sant’Ana A.S. (2021). Biopreservation and probiotic potential of a large set of lactic acid bacteria isolated from Brazilian artisanal cheeses: From screening to in product approach. Microbiol. Res..

[43] Sheahan D.G., Jervis H.R. (1976). Comparative histochemistry of gastrointestinal mucosubstances. Am. J. Anat..

[44] Hanifeh M., Spillmann T., Huhtinen M., Sclivagnotis Y.S., Grönthal T., Hynönen U. (2021). Ex-vivo adhesion of *Enterococcus faecalis* and *Enterococcus faecium* to the intestinal mucosa of healthy beagles. Animals.

[45] Kainulainen V., Tang Y., Spillmann T., Kilpinen S., Reunanen J., Saris P.E., Satokari R. (2015). The canine isolate *Lactobacillus acidophilus* LAB20 adheres to intestinal epithelium and attenuates LPS-induced IL-8 secretion of enterocytes in vitro. BMC Microbiol..

[46] Shehata M.G., Masry S.H., Abd El-Aziz N.M., Ridouane F.L., Mirza S.B., El-Sohaimy S.A. (2024). Probiotic potential of lactic acid bacteria isolated from honeybees stomach: Functional and technological insights. Ann. Agric. Sci..

[47] Musazadeh V., Faghfouri A.H., Zarezadeh M., Pakmehr A., Moghaddam P.T., Hamedi-Kalajahi F., Jahandideh A., Ghoreishi Z. (2023). Remarkable impacts of probiotics supplementation in enhancing of the antioxidant status: Results of an umbrella meta-analysis. Front. Nutr..

[48] Li B., Pan L., Sun J. (2022). Novel probiotic lactic acid bacteria were identified from healthy infant feces and exhibited anti-inflammatory capacities. Antioxidants.

[49] Dos Santos T.A., Rosa A.G., da Silva B., de Medeiros J.M., Fernandes L.D., Uliana A.S., Saito M.E., Yonezawa L.A. (2025). Metabolic and cardiovascular effects of obesity in domestic cats. Vet. Res. Commun..

[50] Telles N.J., Simon B.T., Scallan E.M., Gould E.N., Papich M.G., He Y., Lee M.T., Lidbury J.A., Steiner J.M., Kathrani A. (2022). Evaluation of gastrointestinal transit times and pH in healthy cats using a continuous pH monitoring system. J. Feline Med. Surg..

[51] Jang H., Kim J., Kim Y. (2024). Characterization of feline-originated probiotics *Lactobacillus rhamnosus* CACC612 and *Bifidobacterium animalis* subsp. *lactis* CACC789 and evaluation of their host response. BMC Vet. Res..

[52] Kim T., Mondal S.C., Jeong C.R., Kim S.R., Ban O.H., Jung Y.H., Yang J., Kim S.J. (2022). Safety evaluation of *Lactococcus lactis* IDCC 2301 isolated from homemade cheese. Food Sci. Nutr..

[53] Hussain N., Tariq M., Saris P.E.J., Zaidi A. (2021). Evaluation of the probiotic and postbiotic potential of lactic acid bacteria from artisanal dairy products against pathogens. J. Infect. Dev. Ctries..

[54] Zareie Z., Moayedi A., Garavand F., Tabar-Heydar K., Khomeiri M., Maghsoudlou Y. (2023). Probiotic properties, safety assessment, and aroma-generating attributes of some lactic acid bacteria isolated from Iranian traditional cheese. Fermentation.

[55] Campedelli I., Mathur H., Salvetti E., Clarke S., Rea M.C., Torriani S., Ross R.P., Hill C., O’Toole P.W. (2019). Genus-wide assessment of antibiotic resistance in *Lactobacillus* spp.. Appl. Environ. Microbiol..

[56] Devirgiliis C., Zinno P., Perozzi G. (2013). Update on antibiotic resistance in foodborne *Lactobacillus* and *Lactococcus* species. Front. Microbiol..

[57] Marks S.L., Rankin S.C., Byrne B.A., Weese J.S. (2011). Enteropathogenic bacteria in dogs and cats: Diagnosis, epidemiology, treatment, and control. J. Vet. Intern. Med..

[58] Yao Y., Yang Z., Xie T., Zhang Y., Huang F., Meng C., Wu Y. (2026). Multi-omics analyses of the gut microbiota and metabolism in cats with different body conditions and the effects of fecal microbiota transplantation. Vet. Sci..

[59] Ibrahim S.A., Ayivi R.D., Zimmerman T., Siddiqui S.A., Altemimi A.B., Fidan H., Esatbeyoglu T., Bakhshayesh R.V. (2021). Lactic Acid Bacteria as Antimicrobial Agents: Food Safety and Microbial Food Spoilage Prevention. Foods.

[60] Ji Q.Y., Wang W., Yan H., Qu H., Liu Y., Qian Y., Gu R. (2023). The Effect of Different Organic Acids and Their Combination on the Cell Barrier and Biofilm of *Escherichia coli*. Foods.

[61] Scillato M., Spitale A., Mongelli G., Privitera G.F., Mangano K., Cianci A., Stefani S., Santagati M. (2021). Antimicrobial properties of *Lactobacillus* cell-free supernatants against multidrug-resistant urogenital pathogens. MicrobiologyOpen.

[62] Zhong Y., Lei Y., Jiang S., Chen D., Wang X., Wang K., Liao T., Liao R., Gan M., Niu L. (2025). Advances in understanding the role of gut microbiota in fat deposition and lipid metabolism. J. Anim. Sci. Biotechnol..

[63] Lee D., Kim M., Han J. (2024). GPR41 and GPR43: From development to metabolic regulation. Biomed. Pharmacother..

[64] Liu X., Wang H., Lu Y., Jin G., Fu L., Mao H., Wu Y., Liu P. (2025). Dietary supplementation with yeast cell wall modulates gut microbiota and SCFAs production to improve intestinal health in adult cats. Food Sci. Nutr..

[65] Han B., Liang S., Sun J., Tao H., Wang Z., Liu B., Wang X., Liu J., Wang J. (2024). The effect of Lactobacillus plantarum on the fecal microbiota, short chain fatty acids, odorous substances, and blood biochemical indices of cats. Microorganisms.

