# Supplementary material for: In Vitro Screening and Characterization of Feline-Derived Lactic Acid Bacteria as Potential Probiotic Candidates with Bile Salt Hydrolase Activity and Cholesterol-Removal Capacity
Source: Microorganisms. 2026 Jul 3;14(7):1466. doi: 10.3390/microorganisms14071466 (PMC13414079; doi:10.3390/microorganisms14071466)
Supplement: Supplementary file 1 [file microorganisms-14-01466-s001.zip › microorganisms-4388813-supplementary.pdf]

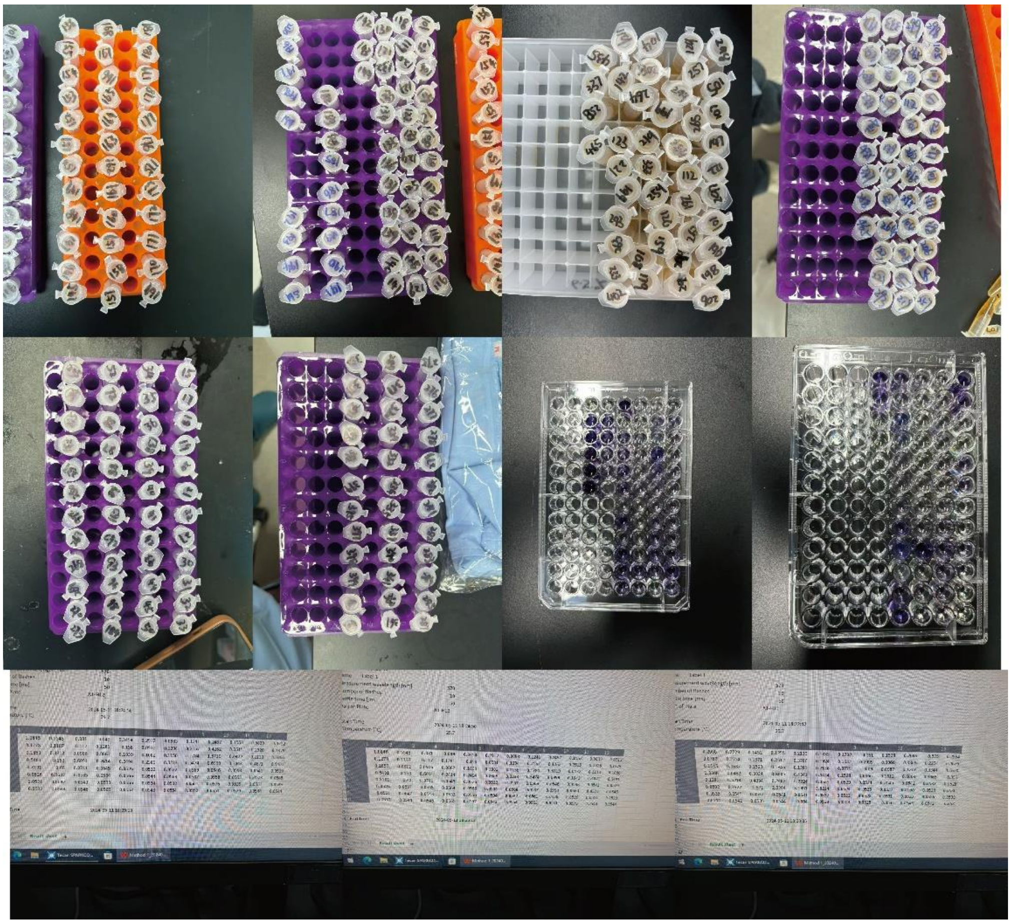

Figure S1. Representative images of the cholesterol-removal assay corresponding to Table 2.

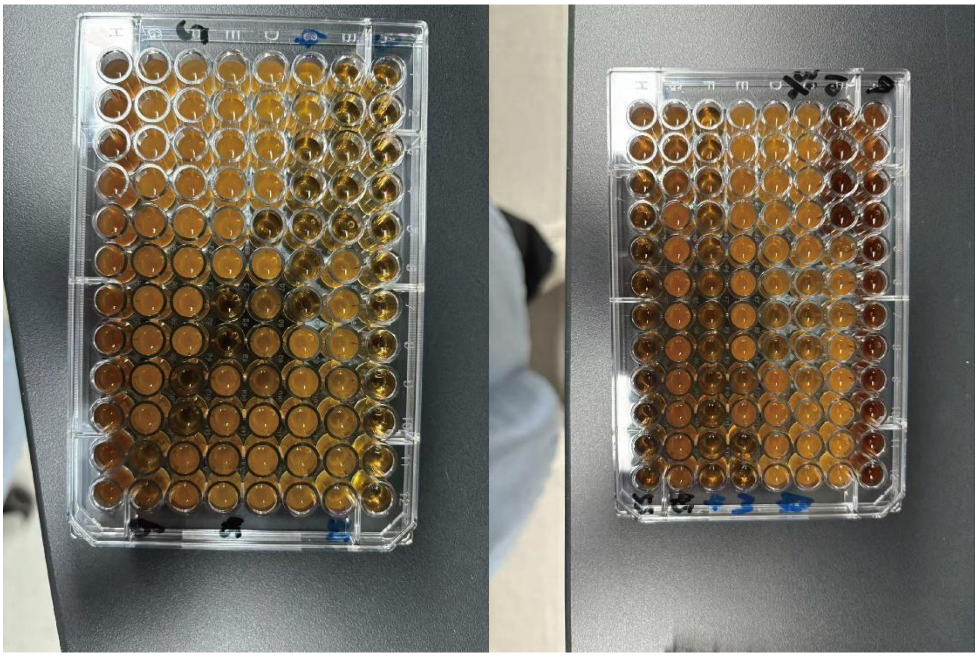

Figure S2. Representative images of the physiological and biochemical characterization assays corresponding to Table 3.

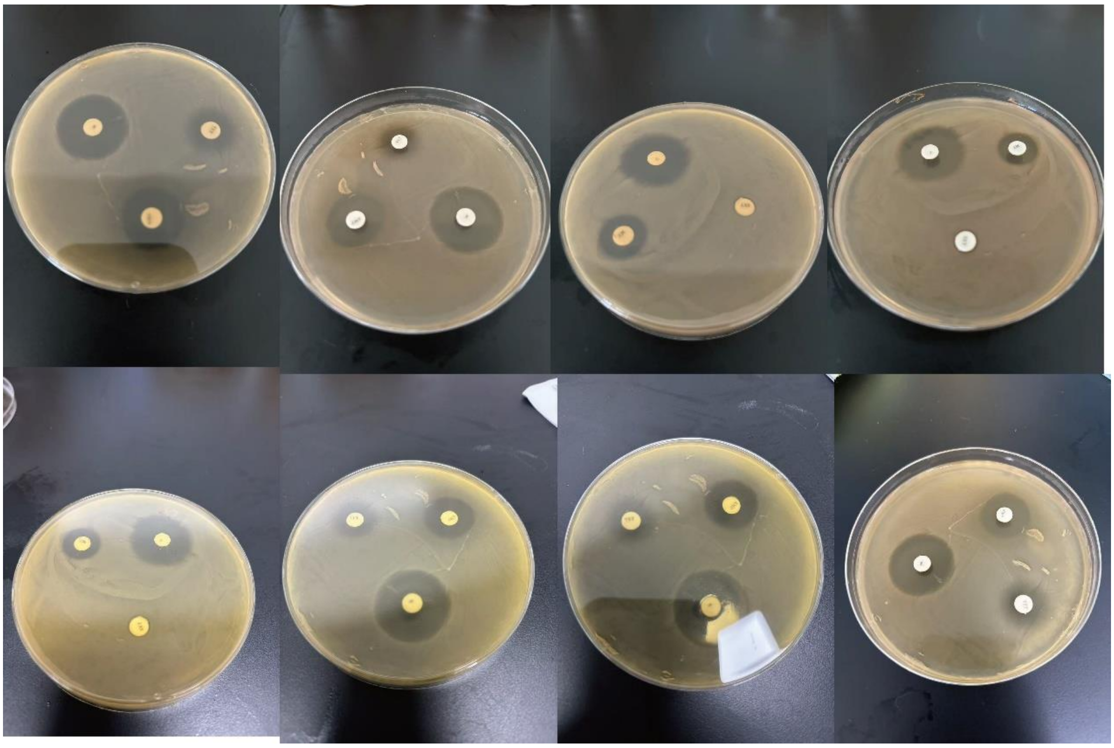

Figure S3. Representative images of the antibiotic susceptibility assay corresponding to Table 4.

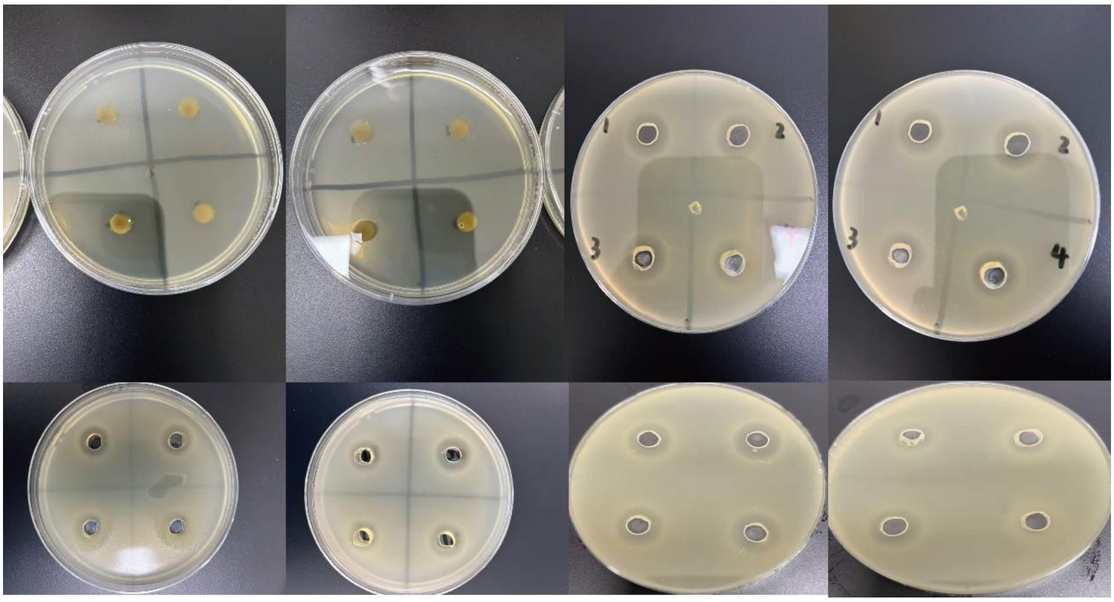

Figure S4. Representative agar plate images of the antibacterial activity assay corresponding to Table 5.

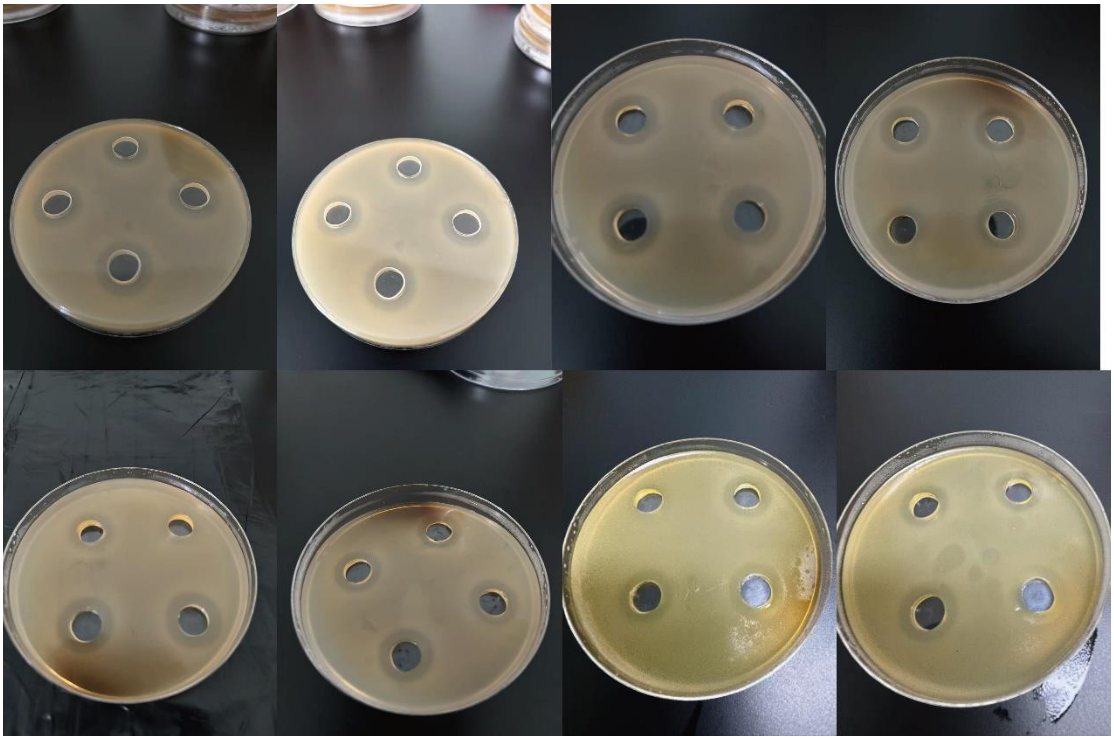

Figure S5. Representative agar plate images showing antibacterial activity after different treatments corresponding to Table 6.
